# Towards a Stochastic Model to Simulate Grapevine Architecture: A Case Study on Digitized Riesling Vines Considering Effects of Elevated CO_2_

**DOI:** 10.3390/plants11060801

**Published:** 2022-03-17

**Authors:** Dominik Schmidt, Katrin Kahlen, Christopher Bahr, Matthias Friedel

**Affiliations:** 1 Department of Modeling and Systems Analysis, Hochschule Geisenheim University, 65366 Geisenheim, Germany; katrin.kahlen@hs-gm.de (K.K.); christopher.bahr@hs-gm.de (C.B.); 2 Department of General and Organic Viticulture, Hochschule Geisenheim University, 65366 Geisenheim, Germany; matthias.friedel@hs-gm.de

**Keywords:** *Vitis vinifera*, functional-structural plant models, Bayesian statistics, model selection, VineyardFACE

## Abstract

Modeling plant growth, in particular with functional-structural plant models, can provide tools to study impacts of changing environments in silico. Simulation studies can be used as pilot studies for reducing the on-field experimental effort when predictive capabilities are given. Robust model calibration leads to less fragile predictions, while introducing uncertainties in predictions allows accounting for natural variability, resulting in stochastic plant growth models. In this study, stochastic model components that can be implemented into the functional-structural plant model Virtual Riesling are developed relying on Bayesian model calibration with the goal to enhance the model towards a fully stochastic model. In this first step, model development targeting phenology, in particular budburst variability, phytomer development rate and internode growth are presented in detail. Multi-objective optimization is applied to estimate a single set of cardinal temperatures, which is used in phenology and growth modeling based on a development days approach. Measurements from two seasons of grapevines grown in a vineyard with free-air carbon dioxide enrichment (FACE) are used; thus, model building and selection are coupled with an investigation as to whether including effects of elevated CO${}_{2}$ conditions to be expected in 2050 would improve the models. The results show how natural variability complicates the detection of possible treatment effects, but demonstrate that Bayesian calibration in combination with mixed models can realistically recover natural shoot growth variability in predictions. We expect these and further stochastic model extensions to result in more realistic virtual plant simulations to study effects, which are used to conduct in silico studies of canopy microclimate and its effects on grape health and quality.

## 1. Introduction

Robust model parametrization is crucial for reasonable predictions, but especially challenging in the field of plant growth modeling, where non-linearities requiring numerous parameters are in conflict with sparse data availability [1,2]. To address this dilemma one can control for and preserve sources of variability in model building, build up on previous knowledge and transport uncertainties into the predictions. Using Bayesian hierarchical models, the aforementioned aspects can be approached in exchange for higher computational demands compared to traditional frequentist approaches [2,3]. In this context, Little [4] suggests frequentist models for model development and checking and Bayesian models for inference. Bayesian hierarchical models were found to be underutilized in the field of ecology a few years back [5], but recently gained more popularity [6]. The benefits of Bayesian hierarchical models include capturing realistic confidence intervals allowing for valuable predictions resembling natural variability [7,8]. While, for instance, crop growth modelers frequently utilize Bayesian models [9,10], they are still rare in specialty crops research, e.g., in grapevine research. Recently, Ellis et al. [11] developed a model to predict grape yield with a double-sigmoid growth model, and Schmidt et al. [12] incorporated Bayesian predictive uncertainties in a functional-structural plant (FSP) model called Virtual Riesling, but mainly limited to modeling the phenological aspect of budburst depending on growing-degree-days (GDD). Since the prediction of phenology and in particular budburst is a prerequisite for modeling plant growth to determine the starting point of vegetative growth, there are several studies that focus exclusively on improving phenological models.

### 1.1. Modeling Phenology

An advancement of key phenological stages due to rising temperatures has already been observed in viticulture, especially in cool-moderate climates [13,14]. As this trend is expected to continue, phenological modeling is regularly applied in studying effects of climate change [15,16,17,18]. In viticultural research there are already several models available predicting various phenological stages, especially budburst and flowering, using recent and historical as well as local and global data for model calibration (e.g., [19,20,21,22,23]). Model accuracies usually allow predictions of major stages within ±7 days [24,25,26,27], while later stages characterizing berry maturity have shown to be less predictable only using temperature data as input [27]. These phenological models are based on growing-degree-days approaches or equivalents, thus relying on temperature response functions that attribute a development contribution to each day depending on daily or hourly summary measures of local air temperatures. Calibration then includes the estimation of threshold temperatures and the necessary sum of thermal development contribution, i.e., GDD, to reach a specific stage or to transfer from one stage to another [25,28]. Research on phenological models includes considerations of various shapes of the response function and on the resolution of input data [24,25,29,30], where sub-daily temperatures and non-linear responses that have also been proven beneficial in crop modeling [31] become more prominent in grapevine phenology modeling, too. [28,30,32,33]. Thus, usually more parameters need to be estimated which is sometimes counteracted by limiting the search space by fixing parameters to reasonable values [19,28,32]. To consider different grapevine varieties either an entire calibration for each variety is used [28] or universally applicable temperature thresholds are combined with variety-specific accumulated development contributions [24]. Further details concerning different modeling approaches are given in Appendix A.1.

### 1.2. Plant Growth Modeling

Phenological development is closely linked to general plant growth as the phenological stages represent appearances and dimensions of plant organs. For instance, grapevine growth stages of the so called modified E-L-system [34,35] include stages such as stage 12: “5 leaves separated; shoots about 10 cm long; inflorescence clear” [34]. Hence, it is reasonable to assume that plant growth and organ development, at least to some extent, can also be modeled with a growing-degree-days approach. Therefore, several empirical growth models incorporating GDD have been developed in the past for numerous plants. While sometimes criticized for their simplicity, empirical models have proven to provide valuable insights [36]. Crop models can be used to predict different growth stages and yield (e.g., [37,38,39,40]), while other plant models also aim to model the architectural development, i.e., the dimensions of plant organs or phytomer development [41,42,43], especially in the case of functional-structural plant (FSP) models (e.g., [44]). Focusing on the parametrization of FSP models, it is of particular interest to simulate an accurate representation of the plant’s three-dimensional architecture over time, if micro-climatic conditions within the canopy are of interest. This can be, for example, local light interactions or derived measures, such as leaf or fruit surface temperatures [45,46,47,48,49,50]. For grapevine, it was found that leaf area and shoot growth can be related to growing degree days [12,42].

Implementing some variability of the plants architecture in FSP models is common practice to counteract artifactual effects that could lead to biased predictions [47,51,52]. Therefore, several approaches have been applied: Parameters known to be non-constant can be randomly sampled from a uniform distribution, e.g., the initial plant orientation of a greenhouse grown cucumber [53]. If there is more information on the distribution of the parameter value, such as its standard deviation estimated by a statistical model or a probability density function based on measurement data, sampling from any given distribution could be applied [12,54]. Depending on the depth of architectural information, a bounding box can be filled with randomly distributed leaves to reconstruct a canopy, with or without neglecting branching structures [45,55], or the dynamic architectural development of a single shoot can be simulated [12]. Both budburst and shoot growth can be strongly heterogeneous within individual grapevines, especially when cane-pruned, due to the influence of acrotony [56]. Bayesian models offer the chance to reproduce such heterogeneous distributions.

### 1.3. Bayesian Model Calibration

Estimating parameter distributions directly from measurements not only requires a specific measurement of this parameter, e.g., measuring leaf inclination angles, but also requires these measurements to be independent samples. To control for deviations from independence, such as repeated measures, statistical models, in particular mixed models, are applicable [57,58,59,60,61]. However, if model parameters need to be estimated that cannot be measured directly, e.g., growth rates, a suitable model function must first be determined, e.g., a logistic growth model, that allows the estimation of such auxiliary model parameters. When estimating these parameters with classical frequentist models, including least squares or maximum likelihood approaches, usually best fit parameters and their empirical confidence intervals are returned. If a Bayesian modeling approach is used instead, entire sets of plausible parameter estimates are returned, where each parameter can be summarized by a posterior probability distribution [62,63]. At first glance, this does not seem to be too different from the output of a frequentist model that also includes parameter uncertainties. However, in a Bayesian framework the sets of parameters included in the non-summarized output, allow multiple independent predictions. Simple frequentist model predictions use the point estimates of the parameters that can only be accompanied by an empirical confidence interval. This advantage of multiple plausible model parameter combinations can be transported in plant growth models in such a way that for each new simulation of an organ or plant, we can sample from the pool of possible parameter combinations to resemble natural variability in repeated simulations. In this way, estimated parameter uncertainty can be transported into model output uncertainties. The strength of Bayesian calibration providing sets of parameters is especially beneficial in multiple parameter models, where dependencies between variables are retained in the sampling. Single parameter estimates that allow sampling from normal distributions based on mean estimates and frequentist confidence intervals or Bayesian credible intervals should be very similar when flat priors and a Gaussian likelihood is used in the Bayesian model. Here we can see another possible advantage of using a Bayesian framework for model calibration. If there is prior information available, we can incorporate this knowledge and update our prior belief on parameter probability distribution with new data [64,65]. This can lead to narrower uncertainties in the output conditional of our data. In summary, by estimating uncertainties within the model parameters and not only in the output, we can repeatedly sample from random trajectories and, hence, conduct stochastic simulations [66].

If clusters or repeated measures are included in the data sampling process, so called mixed or hierarchical models are necessary [59,60,61,63]. Especially in complex models, Bayesian models can outperform classical maximum likelihood estimations by their direct treatment of such conditional structures [57]. Using Bayesian mixed models to calibrate plant growth models is a way of respecting the fact that input information will never be “sufficiently accurate and comprehensive to allow exact inference of parameter values” [2]. Still today, plant (growth) models incorporating Bayesian models in the calibration, and in particular mixed models, are rare. Yet, a recent increase in popularity might be related to and further promoted by recent developments in user-friendly access to Bayesian model building via open-source R or Python packages such as rstan [67], rstanarm [68], brms [69,70] or python PyMC3 [71], bambi [72], an increase in computational resources [65] as well as an increase in introductory textbooks [73,74,75].

In Jansen and Hagenaars [64] general potentials of of Bayesian model calibration are theoretically discussed, especially the concept of consecutively updating the model when new data are available. Oijen et al. [2] introduced Bayesian model calibration for process-based forest models to address issues of reliability in complex model calibration with the benefit of quantifying output uncertainties. Bayesian calibration approaches have also been successfully applied in other fields, especially to benefit from enriched information on parameter uncertainties, the ability to transport these uncertainties in predictions or the incorporation of prior information [76,77,78,79,80,81,82,83] (see also Appendix A.2). While Bayesian methods have in general been proven beneficial for model calibration, they were still underrepresented as shown in a recent study on comparing calibration practices of different crop modeling groups [84], with a similar frequency of use as found in an earlier survey [85].

In the field of viticulture research, Ellis et al. [11] developed a Bayesian model for grapevine yield prediction that was subjected to different prior assumptions. They conclude that Bayesian models might be especially interesting for viticultural models, as predictive capabilities might benefit from the ability of incorporating prior knowledge on vineyard specifics that could be provided by local experts. Recently, Parker et al. [86] conduced a Bayesian study on inflorescence and flower counts as well as on flowering progression, where, for example, high variability in flowering progression was found not only between seasons but also between inflorescences within a season.

In this study, we aim to show the first steps of an advanced robust Bayesian model calibration, paving the way for a fully stochastic version of the functional-structural plant model Virtual Riesling by Schmidt et al. [12]. In the process, empirical model complexity is reduced where justified [87]. To let the reader participate in the development process, decisions made are discussed in more detail than usual. We incorporate novel data from an additional vegetation period and exemplary demonstrate how main parts of the original model, especially budburst prediction and internode development and growth at primary shoots, can be improved and extended by Bayesian model calibration. Novel data now also include observations from plants grown under elevated CO${}_{2}$ (eCO${}_{2}$) conditions for two seasons, hence allowing further model extensions to estimate possible effects of eCO${}_{2}$ on budburst and internode growth. We demonstrate how the flexible Bayesian modeling structure, provided by the brms-package, allows us to estimate the complex hierarchical data structure even with hard-to-estimate non-linear empirical models, leading to more consistent results across different growing seasons, while covering the high natural variability.

## 2. Materials and Methods

### 2.1. Experimental Site

The study was conducted on *Vitis vinifera* L. cv. Riesling vines (clone 198-30 Gm grafted on rootstock SO4 clone 47 Gm) grown in the VineyardFACE system in Geisenheim, Germany. In brief, the free air CO${}_{2}$ enrichment (FACE) system consists of six rings with ambient (aCO${}_{2}$) or elevated CO${}_{2}$ (eCO${}_{2}$) concentrations (20% enrichment, at the time of the experiments roughly 480 ppm) to the grapevines in threefold replicates [88]. Data was gathered in two years (2018, 2019) and includes phenological ratings of randomly selected shoots from almost all Riesling vines of the facility and data on primary shoot growth (number of nodes and internode length) from manual 3D digitization of six selected vines (one vine per ring) using a electromagnetic field digtizer (Fastrak^®^, Polhemus, Colchester, VT, USA). Three digitizations per vine were conducted each year before shoot trimming. Hourly air temperature data was provided by a weather station located at the VineyardFACE site. More details on the FACE facility and the digitization procedure can be found in Wohlfahrt et al. [88] and Schmidt et al. [12], respectively.

### 2.2. Cumulative Development Days Model

In order to account for non-linear responses of growth and development to ambient temperatures, we decided to use the non-linear method introduced by Wang and Engel (Equation (6) [29]) and recently applied for grapevine by Zhu et al. (Equations (3)–(4) [28]). Non-linearity in temperature response is achieved by calculating the hourly development day contribution (hDD) based on a beta-distribution-like function depending on the average hourly air temperature $T_{h}$ (${}^{\circ }C$) and three additional parameters. The latter are the three cardinal temperatures, the base (minimum) temperature ($T_{\mathrm{base}}$, ${}^{\circ }C$), the optimal temperature ($T_{\mathrm{opt}}$, ${}^{\circ }C$) and the upper limit temperature ($T_{\mathrm{upper}}$, ${}^{\circ }C$) for growth. The adopted temperature response function for estimating hourly development day contribution was defined as follows:

(1) $$\mathrm{hDD}=\left\{\begin{array}{cc} 0 & T_{h}<T_{\mathrm{base}}\\ \frac{2{\left(T_{h}-T_{\mathrm{base}}\right)}^{\alpha }{\left(T_{\mathrm{opt}}-T_{\mathrm{base}}\right)}^{\alpha }-{\left(T_{h}-T_{\mathrm{base}}\right)}^{2\alpha }}{{\left(T_{\mathrm{opt}}-T_{\mathrm{base}}\right)}^{2\alpha }}\; & T_{\mathrm{base}}\leq T_{h}\leq T_{\mathrm{upper}}\\ 0 & T_{\mathrm{upper}}<T_{h}\;, \end{array}\right.$$

with

(2) $$\alpha =\ln\left(2\right)/\ln\left[\left(T_{\mathrm{upper}}-T_{\mathrm{base}})/(T_{\mathrm{opt}}-T_{\mathrm{base}}\right)\right]\;,$$

restricting hDD to the values between 0–1. The daily development day contribution DD is then estimated as the average hDD for a day ( 24 h):

(3) $$\mathrm{DD}\left(\mathrm{doy}\right)=\left(\sum_{i=1}^{24}{\mathrm{hDD}}_{\mathrm{doy},i}\right)/24\;.$$

Similar to a growing degree days accumulation, cumulative development days (CDD) are the sum of the development days DD between a starting day (doy${}_{0}$) and the current day of the year (doy):

(4) $$\mathrm{CDD}\left(\mathrm{doy}\right)=\sum_{n={\mathrm{doy}}_{0}}^{\mathrm{doy}}\mathrm{DD}\left(n\right)$$

The maximum hourly contribution of hDD=1 is reached if $T_{h}=T_{\mathrm{opt}}$, while no contribution is accounted for hours with $T_{h}<T_{\mathrm{base}}$ or $T_{h}>T_{\mathrm{upper}}$. Consequently, we expect single day values of DD to be ≪1, as only 24 h of $T_{h}=T_{\mathrm{opt}}$ would yield DD=1.

In contrast to Zhu et al. [28], we do not need to interpolate hourly data from daily summary measures, as we use hourly average air temperatures from the on-site weather station.

### 2.3. Grapevine Phenology Assessment

**Figure 1:**
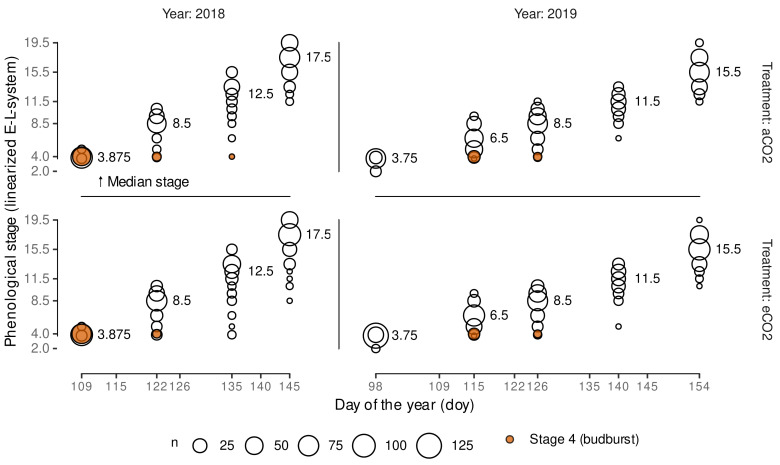
Phenological ratings converted to a linearized modified E-L-system (Table 1) based on single shoots for two different years and the two treatments (ambient end elevated CO${}_{2}$ conditions). Budburst is highlighted, as it marks the starting date for the plant growth model. The bubble size indicates the respective number of observations (*n*). The numbers to the right of a bubble stream indicate the median stage at that time.

Estimation of the three cardinal temperatures for the development days method should include the phenological development. Therefore, grapevine phenology of up to 60 randomly selected shoots (Figure 1, Table A1) in each VineyardFACE ring was assessed by viticultural experts on four and five dates during the 2018 and 2019 growing season, respectively, using the modified E-L-system (ELSt) [35]. The ratings cover stages 1 (“winter bud”) to 18 (“14 leaves separated; flower caps still in place, but cap colour fading from green”) including two major stages “budburst” (stage 4) and “shoots 10 cm, inflorescence clear, 5 leaves separated” (stage 12) [35].

### 2.4. Grapevine Phenology Linearization

The modified E-L-system does include several stages clearly characterized by the number of leaves separated starting from stage 7 (“first leaf separated from shoot tip“) until stage 18 (“14 leaves separated“). Assuming the early stage organ development rate is constant when relating to CDD we linearized the modified E-L stages to set up a linear model of CDD depending on E-L stage, i.e., a model to estimate the accumulated CDD to reach a specific phenological stage. Our linearization assumes a difference of one stage between each leaf separation starting from stage 7 (Table 1). Fixing ELSt = 4 (budburst) to ELSt${}_{\mathrm{linear}}$ = 4 we assume ELSt = 7 (first leaf separated) to be approximated with ELSt${}_{\mathrm{linear}}$ = 6.5, hence not to far off from the original stage number and based on the modeling experiences from Schmidt et al. [12], where budburst was predicted using early E-L stages without linearization. Stages before budburst were drawn closer together and ELSt = 5 remained ELSt${}_{\mathrm{linear}}$ = 5. The full linearization scheme for ELSt 1-18 to linearized scale ELSt${}_{\mathrm{linear}}$ is given in Table 1. This linearization based on organ development should not only allow us to use a linear model relating CDD to ELSt${}_{\mathrm{linear}}$, it is also useful to synchronize organ development rate estimates using phenological and morphological measures in the estimation of cardinal temperatures.

### 2.5. Estimation of Cardinal Temperatures by Multi-Objective Optimization

Our first goal is to obtain robust estimates of the cardinal temperatures $T_{\mathrm{base}}$, $T_{\mathrm{opt}}$ and $T_{\mathrm{upper}}$ that allow us the prediction of necessary CDD for budburst and its variability for a given year, as this is to be used as a starting point for organ development in the plant growth model. In addition, these estimates should also serve as the base for all other temperature dependent growth processes. This is partly in contrast to Zhu et al. [28], where budburst was separated from the general phenology module. We assume that considered phenology stages and temperature dependent growth processes can be predicted with an equal response to temperature, which is in agreement with the results of a meta-analysis of Parent and Tardieu [89]. However, this implies that in particular our budburst predictions will not use information on chilling units resembling an endo-dormancy phase, although this has been considered in other phenological models for grapevine [25,28]. For a robust estimation of the cardinal temperatures all observed linearized stages (Table 1) are considered equally in the calibration. We assume that CDD accumulation starts on 1 January (${\mathrm{doy}}_{0}=1$) and necessary CDD to reach a specific stage is constant across years, hence, dependent on air temperature, but it might also be affected by the treatment, i.e., eCO${}_{2}$ or aCO${}_{2}$.

A linear mixed model was built to predict CDD depending on ${\mathrm{ELSt}}_{\mathrm{linear}}$, treatment ($\mathrm{trt}\in \{{\mathrm{eCO}}_{2},{\mathrm{aCO}}_{2}\}$) and year (2018, 2019; categorical variable) plus the interaction of $\mathrm{trt}\times {\mathrm{ELSt}}_{\mathrm{linear}}$. In the mixed model part we control for repeated samplings in rings over the years and on single plants within a year (rankings of multiple shoots from the same vine) using group-level effects on the intercept. In addition, we allowed the slope to vary between plants. This results in the following model formulation according to the lme4/brms-package syntax [70,90]:

(5) $$\mathrm{CDD}∼\mathrm{trt}\times {\mathrm{ELSt}}_{\mathrm{linear}}+\mathrm{year}+\left(1|\mathrm{ring}\right)+\left(1|\mathrm{year}:\mathrm{ring}\right)+\left(1|\mathrm{plant}\right)+\left(0+{\mathrm{ELSt}}_{\mathrm{linear}}|\mathrm{plant}\right)\;.$$

For hold-out validation, the data was split into a training and a test data set before model fitting using a ratio of approx. 80%/20% while blocking by year, treatment and plant, i.e., all data from the same plant within a year is assigned either to the training or the test data ($n_{\mathrm{train}}=2085$, $n_{\mathrm{test}}=599$).

To incorporate also digitization data in the cardinal temperature estimation, we use the information on the development of the highest rank from each primary shoot, i.e., the rank of the apex ($R_{\mathrm{apex}}$). Similar to Schmidt et al. [12], we use the apex rank to estimate an internode appearance rate (IAR, ranks/CDD) by relating $R_{\mathrm{apex}}$ to CDD. We assume a linear dependency between apex rank ($R_{\mathrm{apex}}$) and CDD during the time frame of our observation (maximum observed $R_{\mathrm{apex}}$ = 23). For the cardinal temperature estimation a linear mixed model (Equation (6)) was set up, where we were mainly interested in the slope parameter of the model representing the IAR. The model includes fixed effects for CDD, year and treatment, plus the interaction of CDD×trt, while considering group-level effects of the ring, the plant, i.e., shoots from the same plant in a year, and the shoot, i.e., repeated measures from a single shoot, plus a varying slope for each shoot.

(6) $$R_{\mathrm{apex}}∼\mathrm{CDD}\times \mathrm{trt}+\mathrm{year}+\left(1|\mathrm{ring}\right)+\left(1|\mathrm{plant}\right)+\left(1|\mathrm{shoot}\right)+\left(0+\mathrm{CDD}|\mathrm{shoot}\right)\;.$$

Furthermore, here, data was split into a training and a test data set (approx. 80%/20% split) with blocking by treatment, year and shoot ($n_{train}$ = 200, $n_{test}$ = 56).

By including two different data sets into the cardinal temperature estimation, we aim for more robust estimates, allowing the prediction of budburst and its variability and the organ (internode) development. Based on our assumptions, both models are linked by their slope parameters representing organ development rates. The inverse of the slopes for each treatment of Equation (6), i.e., the IAR in ranks/CDD should be equal to the slopes from Equation (5), which describes the leaf appearance rate (LAR) as CDD/leaf per treatment.

In order to estimate parameters that allow predicting corresponding CDD for growth stages and treatments independent of the year but with similar organ development rates in both models, we set up a multi-objective optimization [91]. Its objectives were to simultaneously minimize the

- treatment-averaged normalized absolute difference between the organ development rates ($\Delta \bar{ODR}$)
- absolute effect of year in both models ($E_{\mathrm{year},\mathrm{phen}}$,$E_{\mathrm{year},\mathrm{apex}}$)

while constraining the normalized root-mean squared errors for both models for both the test and the training data (${\mathrm{NRMSE}}_{\mathrm{train},\mathrm{phen}}$, ${\mathrm{NRMSE}}_{\mathrm{test},\mathrm{phen}}$, ${\mathrm{NRMSE}}_{\mathrm{train},\mathrm{apex}}$, ${\mathrm{NRMSE}}_{\mathrm{test},\mathrm{apex}}$) with

(7) $$\Delta \bar{ODR}=\left(\frac{|\frac{1}{{\mathrm{IAR}}_{\mathrm{aCO}2}}-{\mathrm{LAR}}_{\mathrm{aCO}2}|}{(\frac{1}{{\mathrm{IAR}}_{\mathrm{aCO}2}}+{\mathrm{LAR}}_{\mathrm{aCO}2})/2}+\frac{|\frac{1}{{\mathrm{IAR}}_{\mathrm{eCO}2}}-{\mathrm{LAR}}_{\mathrm{eCO}2}|}{(\frac{1}{{\mathrm{IAR}}_{\mathrm{eCO}2}}+{\mathrm{LAR}}_{\mathrm{eCO}2})/2}\right)/2\;,$$

and

(8) $${\mathrm{NRMSE}}_{\mathrm{phen}}=\frac{\sqrt{\sum_{i=1}^{n}}{\left(\mathrm{CDD}-\hat{\mathrm{CDD}}\right)}^{2}}{\bar{\mathrm{CDD}}}\;,$$

and

(9) $${\mathrm{NRMSE}}_{\mathrm{apex}}=\frac{\sqrt{\sum_{i=1}^{n}}{\left(R_{\mathrm{apex}}-\hat{R_{\mathrm{apex}}}\right)}^{2}}{\bar{R_{\mathrm{apex}}}}\;,$$

where the CD^ indicates the predicted values.

The parameter estimation was conducted using a step-wise grid search approach [92]. The initial grid search used step sizes of 1 ${}^{\circ }C$ for all parameters and was set up with $T_{\mathrm{base}}\in \left\{-60,-59,-58,\cdots ,15{\;}^{\circ }C\right\}$, $T_{\mathrm{opt}}\in \left\{10,11,12,\cdots ,35{\;}^{\circ }C\right\}$ and $T_{\mathrm{upper}}\in \left\{15,16,17,\cdots ,55{\;}^{\circ }C\right\}$. To account for overlaps in temperature intervals we only allowed $T_{\mathrm{base}}\leq T_{\mathrm{opt}}-2{}^{\circ }C$ and $T_{\mathrm{opt}}\leq T_{\mathrm{upper}}-2{}^{\circ }C$ within a parameter set of $T_{\mathrm{base}}$, $T_{\mathrm{opt}}$ and $T_{\mathrm{upper}}$ leading to a total of 37,778 combinations.

In the second and final iteration a refined grid search with 6858 additional combinations around the estimated minimum was conducted using the following parameter ranges and step sizes: $T_{\mathrm{base}}\in \left\{10.1,10.2,10.3,\cdots ,11.9\right\}$, $T_{\mathrm{opt}}\in \left\{18.1,18.2,18.3,\cdots 19.9\right\}$, $T_{\mathrm{upper}}\in \left\{24.1,24.2,24.3,\cdots ,25.9\right\}$. We refrained from lowering the step size any further or applying full range classical global optimization algorithm, as the expected measurement accuracy of future input data—average hourly measurements of air temperature from local weather stations—would not justify any further refinement.

Following each iteration the optimum was selected from a set of Pareto optimal solutions [91] estimated on a subset that only included potential optima by using thresholds on the output parameters given the following conditions:

$$\begin{array}{cc} \Delta \bar{ODR} & <0.05\;\mathrm{and}\\ E_{\mathrm{year},\mathrm{phen}} & <0.05\;\mathrm{and}\\ E_{\mathrm{year},\mathrm{apex}} & <0.05\;\mathrm{and}\\ {\mathrm{NRMSE}}_{\mathrm{train},\mathrm{phen}} & <0.20\;\mathrm{and}\\ {\mathrm{NRMSE}}_{\mathrm{train},\mathrm{apex}} & <0.20\;\mathrm{and}\\ |{\mathrm{NRMSE}}_{\mathrm{train},\mathrm{phen}}-{\mathrm{NRMSE}}_{\mathrm{test},\mathrm{phen}}| & <0.05\;\mathrm{and}\\ |{\mathrm{NRMSE}}_{\mathrm{train},\mathrm{apex}}-{\mathrm{NRMSE}}_{\mathrm{test},\mathrm{apex}}| & <0.05\; \end{array}$$

For the final optimization step the three main parameters $\Delta \bar{ODR}$, $E_{\mathrm{year},\mathrm{phen}}$ and $E_{\mathrm{year},\mathrm{apex}}$ addressing our assumptions on similar organ development rates across data sets and no effect of the year were scaled to the range of 0–1 using min-max normalization following:

(10) $${\mathrm{value}}_{01}=\frac{\mathrm{value}-\min}{\max-\min}\;.$$

The previously applied thresholds assured adequate predictive accuracy for combinations within the subset; hence, we did not need to include NRMSE-values here.

The set of Pareto optimal solutions was created using a a weighted sum approach following

(11) $$f\left(w_{1},w_{2},w_{3}\right)=\;w_{1}\;\cdot \;\Delta \bar{ODR}+w_{2}\;\cdot \;E_{\mathrm{year},\mathrm{phen},01}+w_{3}\;\cdot \;E_{\mathrm{year},\mathrm{apex},01}\;,$$

with $w_{1}$,$w_{2}$ and $w_{3}\in \left\{0,0.05,0.10,\cdots ,1\right\}$ and $w_{1}+w_{2}+w_{3}=1$. Each of these 219 combinations of weighting factors provides an optimum solution from within the subset associated with a cardinal temperature triplet of $T_{\mathrm{base}}$, $T_{\mathrm{opt}}$, $T_{\mathrm{upper}}$. The overall best cardinal temperature combination was selected as the one most frequently associated with a Pareto optimum solution.

### 2.6. Modeling Budburst Variability

Following the parameter optimization, which was based on a linear mixed model with maximum likelihood estimation, the budburst model was fit in a Bayesian framework. This Bayesian calibration not only allowed predicting a mean CDD for the linearized E-L-system growth stage 4 (budburst), using posterior predictions, information on CDD variability could also be estimated. The initial Bayesian calibration was followed by a step-wise model reduction to evaluate whether the factor year can be fully excluded as assumed and whether there is a treatment effect on phenological development. Our decisions were based on information criteria, hold-out validation performance and, in the case of Bayesian models, the probability of direction [93,94,95]. Bayesian models were compared by the LOOIC (leave-one-out cross-validation (LOO) information criterion) derived from the ELPD (expected log predictive density) based on Pareto smoothed importance sampling [94]. We also provide general performance measures that underline our decision on the final model, i.e., the selected model after model reduction, such as RMSE and (Bayesian) R2 (R-squared). In addition, we computed the probability of direction (pd) for fixed model parameters, which indicates whether a parameter was estimated to be strictly positive or negative, with values between 50% (no direction) and 100% (one direction). Being strongly correlated with *p*-values from frequentist approaches, pd-values of at least 97.5% are necessary to approximately correspond to a two-sided *p*-value of 0.05. In addition, we also refitted frequentist versions of the models for comparison using AICc, a second-order Akaike Information Criterion [96]. In the first step, the $\mathrm{trt}\times {\mathrm{ELSt}}_{\mathrm{linear}}$-interaction was excluded, before the year effect could be removed entirely matching the initial assumption that there is no effect of year on necessary CDD to reach budburst. Finally, we evaluated the effect of removing the trt effect.

Posterior predictive checks of the final model indicated that the assumed Gaussian likelihood might not be the best fit to the data due to its inflexibility in capturing possible skewness. Hence, we refitted the final Bayesian model as a generalized linear mixed model using an exponentially modified Gaussian distribution likelihood (exGaussian) [97]. By calculating the DD and the resulting CDD for each day of the year (doy) model posterior predictions of budburst CDD and its variability can be mapped back to visualize the predicted time frame for budburst within a year.

### 2.7. Modeling Internode Development

To simulate the growth of plant organs, e.g., in a functional-structural plant model, the development of phytomers and the growth of a plant organ must be represented in model functions. As already introduced above and similar to Schmidt et al. [12], we estimate the internode appearance rate (IAR, ranks/CDD) by using information from digitized shoots and relate them to CDD. Besides integrating novel data from a second season and from plants grown under elevated CO${}_{2}$, a further advancement related to Schmidt et al. [12] is that we now use the same non-linear CDD function with cardinal temperatures estimated considering phenological development data and digizitazion data. In the cardinal temperature estimation we used a frequentist linear mixed model, to keep computational times low for the repetitive model fitting procedure outlined above. To derive parameters for stochastical modeling we transferred the model to a Bayesian framework. Hence, first a similar Bayesian linear mixed model with a Gaussian likelihood was set up to predict the apex rank depending on cumulative development days (Equation (6)). Then again, model reduction was performed, starting from the full model (Equation (6)). Moreover, here model selection could rely on training and test data set performances. The reduced final model was refitted using the full data set to extract the posterior distribution to define a sampling distribution for the internode appearance rate (IAR).

### 2.8. Internode Length Model

Schmidt et al. [12] found that primary shoot internode growth depending on temperature can be modeled with a non-linear asymptotic growth model when considering the rank of the internode and its ‘age’ (${\mathrm{CDD}}_{\mathrm{age}}$). The internode age is defined as the CDD accumulated after the respective internode appearance. Similar to Schmidt et al. [12], we determine ${\mathrm{CDD}}_{\mathrm{age}}$ by relying on the estimates for IAR and the rank of the apex at the respective digitization date ($R_{\mathrm{apex}}$). For each internode *I* the current age in CDD is estimated following:

(12) $${\mathrm{CDD}}_{\mathrm{age},I}=\left(R_{\mathrm{apex}}-R_{I}+1\right)/\mathrm{IAR}\;.$$

For the internode length model we rely on the average IAR, although we already estimated some uncertainty by estimating the posterior distribution of IAR with the previous model. However, this uncertainty is confounded with the uncertainty we have in our data by being limited to the apex rank present at the digitization date for estimating ${\mathrm{CDD}}_{\mathrm{age}}$. Hence, we included a general age-correction term in our modeling approach to address at least parts of both issues at once. Details on its implementation will be discussed later in this section (Equation (19)).

In general, model building and selection considered the additional factors year and treatment, as well as accounting for the sampling structure with group-level effects. The age-correction term considers group-level effects for each measurement date of a shoot (shoot × date; cf. Listing A1). In Schmidt et al. (Equation (3) [12]) the non-linear part was modeled with an asymptotic regression model through the origin with two parameters, here denoted *A* and lrc (Equation (13)).

(13) $$\mathrm{IL}=A\;\cdot \;(1-\exp\left(-\exp\left(\mathrm{lrc}\right)\;\cdot \;{\mathrm{CDD}}_{\mathrm{age}}\right))\;.$$

Following Schmidt et al. [12], we assume similar growth behavior between ranks, i.e., same lrc for each rank (*R*), but allowing variable asymptotic values, i.e., maximum internode length for each rank. Instead of fitting a separate downstream model to capture the nature between individual per-rank estimates of the asymptote parameter as in Schmidt et al. [12], the non-linear part of the model was extended to include an implicit modeling of the asymptote dependencies on the rank. This was realized by incorporating the two-step approach from Schmidt et al. [12] into a single model formula. Similar to Schmidt et al. [12], the relation between asymptote and ranks ≤ 7 is generally modeled with a linear function (Equation (14)), although recent observations indicated a possible non-linear behavior for internodes up to rank 7.

(14) $$G_{\mathrm{Asym}}=m_{1}\;\cdot \;\left(R-1\right)+i_{1}\;.$$

This non-linearity might have been approximated with a three-parameter Gompertz function ($y=a\;\cdot \;\exp(-b\;\cdot \;c^{x})$); however, in expectation of high variability within the data, the high flexibility of such exponential functions is a hindrance to Bayesian model fitting when aiming for robust estimates. Nevertheless, to partially include the observations on deviations from linearity for ranks ≤ 7, our novel implementation includes parameters allowing to shift the rank input for the ranks 2 and 7. In contrast to Schmidt et al. (Equation (4) [12]), we replaced *R* by (R−1) (Equation (14)) to later control for a positive restricted intercept equaling the asymptotic internode length at rank 1. For the ranks 2 and 7 we allow for an offset to be estimated by the model ((Equations (15) and (16)) that could correct for slight deviations from linearity at these ranks, to mimic a Gompertz-like behavior. Therefore, we introduce variables $R_{2}$ and $R_{7}$ as

(15) $$\begin{array}{cc} R_{2}\left(R\right) & =\left\{\begin{array}{c} 1\;\mathrm{if}\;R=2\\ 0\;\mathrm{if}\;R\neq 2\;, \end{array}\right. \end{array}$$

and

(16) $$\begin{array}{cc} R_{7}\left(R\right) & =\left\{\begin{array}{c} 1\;\mathrm{if}\;R=7\\ 0\;\mathrm{if}\;R\neq 7\;, \end{array}\right. \end{array}$$

and modify Equation (14) as follows:

(17) $$G_{\mathrm{Asym}}=m_{1}\;\cdot \;\left(\left(R-1\right)+R_{2}\;\cdot \;s_{R2}+R_{7}\;\cdot \;s_{R7}\right)+i_{1}\;.$$

For internodes above rank 7 a repetitive linear dependency that is expected to be related to observations of three morphological distinct phytomers [98,99] is realized as a step function and modeled following the form of Schmidt et al. (Equation (4) [12]):

(18) $$L_{\mathrm{Asym}}=R_{x3}\left(R\right)\;\cdot \;m_{2}+i_{2}\;\mathrm{with}\;R_{x3}\left(R\right)=\left(R+1\right)\;\mathrm{mod}\;3$$

Thus, the complete model to predict internode length, IL (cm), depending on the rank (*R*) and the age of the internode (${\mathrm{CDD}}_{\mathrm{age}}$) was implemented as follows:

(19) $$\begin{array}{ccc} \mathrm{IL}(R,{\mathrm{CDD}}_{\mathrm{age}})= & A\left(R\right)\;\cdot \;\left(1-\exp\left(-\exp\left(\mathrm{lrc}\right)\;\cdot \;\left({\mathrm{CDD}}_{\mathrm{age}}+s_{\mathrm{age}}\right)\right)\right) & \mathrm{with} \end{array}$$

(20) $$\begin{array}{ccc} A(R)= & R_{01}\left(R\right)\;\cdot \;G_{\mathrm{Asym}}+\left(1-R_{01}\left(R\right)\right)\;\cdot \;L_{\mathrm{Asym}} & \mathrm{and} \end{array}$$

(21) $$\begin{array}{cc} R_{01}\left(R\right)= & \left\{\begin{array}{cc} 1\;\mathrm{if}\;R\leq 7\\ 0\;\mathrm{if}\;R>7\;, & \end{array}\right. \end{array}$$

where we also included the above mentioned shift of the calculated ${\mathrm{CDD}}_{\mathrm{age}}$ ($s_{\mathrm{age}}$) to account for uncertainties related to the fact that the appearance of the apex rank must not coincide with the digitization date and the uncertainty in the IAR-estimate (see Section 2.7). As the latter one affects the ${\mathrm{CDD}}_{\mathrm{age}}$ of ranks differently we also model a group-level slope for each shoot × date combination (cf. Listing A1).

For held-out evaluation, the data set consisting of 2658 observations was split into a training (≈80%, $n_{I}=2124$, $n_{shoot}=64$) and a test dataset (≈20%, $n_{I}=534$, $n_{shoot}=16$). To get balanced sample sets we used blocking by year, treatment and intervals of maximum rank (apex rank) of a shoot. Assignment to a sample set was then based on shoot affiliation, i.e., all internodes from the same shoot were assigned to the same data set. A summary of the training and test data sets split into year and average internode length is given in Table A2. Within the training data set 571 aCO${}_{2}$ and 565 eCO${}_{2}$ observations from 2018 and 432 and 556 observations from 2019 were present, respectively. Test data included 154 aCO${}_{2}$ and 130 eCO${}_{2}$ internode measurements from 2018 and 136 and 114 from 2019, respectively.

Preliminary model fitting was conducted using a non-linear mixed effects model (‘nlme’-function, R-package *nlme*, v.3.1-152) [100]. This frequentist implementation uses the concept of restricted maximum likelihood (REML), but allows for Gaussian likelihood only. Preliminary model selection was limited to this approach due to the faster convergence (minutes vs. hours) compared to full Bayesian model implementations. Model selection included the consideration of heteroscedasticity depending on the fitted values and per rank, as we expect higher variability for higher measures. While higher ranks are also associated with longer internodes [12], they might be subjected to additional variability due to measurement errors that might increase with distance to the cane. In the final model for predicting internode length we want to assure only positively restricted outcomes. Therefore, we used a Gamma-likelihood with a log-link in the Bayesian model implementation, taking advantage of the high flexibility offered by the *brms*-package. An upstream analysis had shown that we can obtain meaningful parameters on the non-transformed scale, if the right-hand side is log-transformed, while avoiding model initialization problems associated with the use of an ‘identity’ link function. Following prior predictive checks [101] and incorporating expert knowledge gained from 2018 data [12], moderately informative priors (Table A6) were set to achieve convergence for the complex non-linear model [102]. Furthermore, the full model included controlling for heteroscedasticity by group-level effects for rank, ring, plant, shoot and year on the shape parameter of the Gamma distribution likelihood making use of the so-called distributional model capabilities (https://cran.r-project.org/web/packages/brms/vignettes/brms_distreg.html, accessed on 7 February 2022). The Gamma-likelihood with log-link implicitly models variance increasing with the mean.

### 2.9. Model Implementation and Diagnostics

Model implementation and related data analysis were conducted within R (v.4.1.2) [103]. Linear and generalized linear mixed models with restricted maximum likelihood were implemented using the lme4-package (v.1.1-27.1) [90]. For the non-linear mixed models with heteroscedasticity the ‘nlme’-function from the R-package *nlme* (v.3.1-152) [100] was used. To realize the Bayesian model calibration equivalents and advanced models implementations, especially generalized linear and non-liner mixed modeling (e.g., Gamma likelihood) and heteroscedasticity consideration by distributional modeling, the capabilities of the brms-package (v.2.16.1) [69,70] were utilized. Following prior predictive checks [101] and previous experience [12] weakly to moderately informative priors were applied Table A4, Table A5, Table A6. If there were any warnings on divergent transitions, visual Markow-Chain-Monte-Carlo (MCMC) diganostics were used to rule out that the Hamiltonian Monte Carlo algorithm had real problems fitting a model [101]. Bayesian models were run with 4 (or 6) Markov chains with 4000 samples per chain of which the first 2000 iterations are discarded as burn-in, hence, providing a total of 8000 (or 12,000) samples from the posterior, where more samples (six chains) were only used for the complex internode length models. Bayesian model diagnostics did not indicate any errors, i.e., $\hat{R}$ (Rhat) values were all below 1.01 [104], and effective sample size ratios were well above 0.1 [105]. Posterior predictions for performance measures were usually conducted leaving out the group-level effects, as this allows for the direct comparison of training and test data prediction by RMSE. For performance measures used in model comparisons helper functions from the brms-package (e.g., Bayesian R2 [106]) or bayestestR (v.0.11.0) [107] (e.g., probability of direction [93]) were used. The data.table-package (v.1.14.0) [108] was used for data wrangling tasks and visualizations were created with the ggplot2-package (v.3.3.5) [109].

### 2.10. Model Validation

#### 2.10.1. Phenology Prediction

For external validation of the final model that predicts necessary CDD for observed E-L stages point estimate data on budburst from Riesling grown at three selected locations covering seasons from 2003 to 2021 was used. As Riesling is a grapevine variety especially suited for cool climates the three locations Neustadt an der Weinstraße, Germany, Zeltingen-Rachtig, Germany, and Remich, Luxembourg, are characterized by a cool climate, too. We compared the observed budburst day of the year with the average predicted day of the year. In addition, we present the 50% and 95% highest density intervals from the Bayesian model predictions. Predictive error was estimated in days by calculating RMSE, mean absolute error (MAE) and bias between the observation and the average prediction. The average predicted budburst day was estimated from 4000 predictions based on draws from the posterior distribution not considering group-level effects for each scenario. Each posterior prediction provides a CDD threshold for budburst (${\mathrm{ELSt}}_{\mathrm{linear}}=4$) that is mapped to the corresponding day of the year.

Furthermore, we compare the novel model predictions with average predictions from the budburst variability model from Schmidt et al. [12], where in a first step the budburst date prediction model for Riesling from Nendel [21] is applied (Equation (2) [12]). The predicted date is then combined with the estimated variability in GDD based on VineyardFACE aCO${}_{2}$ data from the year 2018 to also predict 4000 budburst dates. Hence, the two model steps rely on two different GDD estimations (see (Equations (1) and (2) [12])).

For Neustadt and Zeltingen-Rachtig the data sources also included the stage ‘beginning of flowering’, defined in the modified E-L system as ‘about 16 leaves separated; beginning of flowering (first flower caps loosening)’ [35]. Being partly associated with the number of seperated leaves we assigned ${\mathrm{ELSt}}_{\mathrm{linear}}=21.5$ to this phenological stage to allow projections of this adjacent stage (Table 1).

In addition, local budburst data from Geisenheim, Germany, between 1990 and 2009 from Stoll et al. [14] are compared to model predictions to evaluate if an observed advancement of budburst date is captured by model predictions.

Data sources for phenology observation are given in Table 2. Local average hourly air temperature was available from Dienstleistungszentren Ländlicher Raum Rheinland-Pfalz, Agrarmeteorologie Rheinland-Pfalz (https://www.am.rlp.de/, accessed on 7 February 2022) using weather stations Neustadt (1/NW), Zeltlingen (112/ZET), Remich (AGM 009, Luxemburg) and from the German Meteorological Service (Deutscher Wetterdienst, DWD) for a local weather station at Geisenheim (station ID 1580).

#### 2.10.2. Predicting the Apex Rank

For external validation of the estimated appearance rate we rely on a data set on Riesling vines collected in 1986 and 1987 at Geisenheim, Germany, where the plastochron index, i.e., the number of leaves on a shoot, was estimated during the growing season (Figure 8 (‘S-System’) [42]). As each node in a grapevine is associated with a leaf, this index is to some extent similar to the apex rank that was used for estimating the primary shoot internode appearance [113]. Predictions were estimated in a two-step process: First we predicted 4000 expected budburst dates, using the same procedure as described above (Section 2.10.1), here using hourly air temperature data from a local weather station at Geisenheim provided by the German Meteorological Service (Deutscher Wetterdienst, DWD; station ID 1580). Second, starting from each budburst day we accumulated the CDD and multiplied it by the estimated slope of internode development IAR. To include some measure of additional uncertainty into the visualization, we also multiplied by $\mathrm{IAR}\pm \sigma_{\mathrm{IAR}}$, hence, considering the estimated standard deviation. Measurement data (averages) were extracted from the original figure using Webplotdigitizer [114], and, if extractable, the provided error interval representing ±2×SE. Observational data was limited to the time frame before hedging. RMSE and bias between the predictions and the measurements were calculated for each of the 4000 budburst dates and then summarized as an average RMSE for the model; hence, the predicted frequencies of each budburst date are considered in this overall RMSE. These predictions and measures are compared to similarly conducted predictions using the respective model components (budburst prediction, appearance rate) from Schmidt et al. [12].

#### 2.10.3. Shoot Length Predictions

External data on shoot length, or even more detailed on internode length, accompanied by high resolution local air temperature data is rare, so we refer to published data from a study of Pagay et al. [115], in which Riesling growth was monitored focusing on water stress effects. In addition, we can rely on local data from preliminary results on shoot length development over time from the Geisenheim VineyardFACE gathered in 2020.

Pagay et al. [115] selected shoots from two different shoot length classes (short, long) and found no effect on growth pattern between rainfed and stressed conditions. The experiment took place at New York State Agricultural Experiment Station, Geneva, NY, USA. In contrast to the Geisenheim VineyardFACE Riesling, which uses *Vitis vinifera* L. cv. Riesling (clone 198-30 Gm) grafted on rootstock SO4 (clone 47 Gm), Pagay et al. [115] used *Vitis vinifera* L. cv. Riesling (Cl. 239) grafted onto 101-14 rootstock. In addition, there are some differences in vineyard setup, for example 8–10 shoots per 90 cm at Geisenheim [12] versus approx. 15 shoots per linear meter of canopy in the study of Pagay et al. [115]. The corresponding high resolution thermal data was provided by personal communication. We compared their data to model predictions for the year 2012. Model predictions were limited to the time frame from average budburst date to accumulated CDD after budburst of ≤30 similar to the time frame of model calibration.

The prediction workflow used for apex rank predictions (cf. Section 2.10.2) was extended by predictions of shoot length, i.e., the sum of internode length of a shoot at a given time. For each predicted budburst date with a frequency of at least 1% (i.e., 40/4000 samples) 80 random shoots and their internodes were simulated with parameter sets drawn from the posterior distribution of lrc, $m_{1}$, $i_{1}$, $m_{2}$, $i_{2}$, $s_{R2}$, $s_{R7}$. group-level effects are sampled for lrc, $m_{1}$, $i_{1}$, $m_{2}$, $i_{2}$. The age correction is fixed to $s_{\mathrm{age}}=0$, as it was intended as a correction factor for the measurements, only. In addition, we simulate budburst and shoot growth using the respective model formulas from Schmidt et al. [12]. Here, only a single shoot per budburst date is simulated from their frequentist internode length model. As the observational data from Pagay et al. [115] does represent selected shoots of different length classes, we only conducted a qualitative comparison between the models and the observation.

For comparison with the local data, similar predictions were computed using Geisenheim local air temperatures from 2020-season.

Finally, we also predicted shoot length for 2018 and 2019 to compare with the calibration data, which was supplemented by measurements from 2019 of unpruned shoots (‘nearby random shoots’) from identical planting material grown outside of the FACE rings [116].

### 2.11. Flowchart of the Model Development and Validation Progress

**Figure 2:**
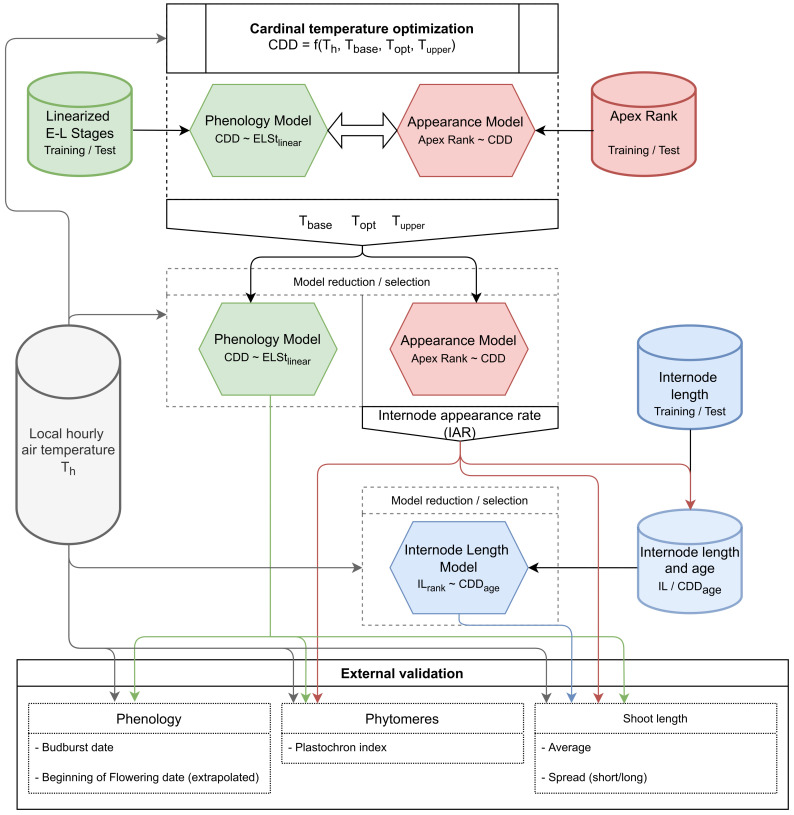
Flowchart on the primary model development and validation steps. Abbreviations: CDD: Cumulative development days; ${\mathrm{ELSt}}_{\mathrm{linear}}$: Linearized phenological Eichorn-Lorenz (E-L) stages [110]; $T_{h}$: Hourly air temperature; $T_{\mathrm{base}}$, $T_{\mathrm{opt}}$, $T_{\mathrm{upper}}$: Cardinal temperature thresholds of non-linear response function (Equation (1)); ${\mathrm{IL}}_{\mathrm{rank}}$: Internode length at a specific rank; ${\mathrm{CDD}}_{\mathrm{age}}$: Age of internode in CDD).

The flowchart (Figure 2) summarizes the previously described general process and the interplay between the developed models in the model predictions. The first block represents the cardinal temperature optimization (see Section 2.5) which is based on the two models for phenology and for apex rank. The resulting temperature triplet ($T_{\mathrm{base}}$, $T_{\mathrm{opt}}$, $T_{\mathrm{upper}}$) is the start point for the model reduction process for both the phenology and the appearance model. The phenology model is later applied to predict budburst dates in all validation scenarios, while the appearance model output is necessary to extend the internode length dataset with an estimate of internode age in CDD. Finally, the internode length model is also subjected to model reduction and selection. External validation on the number of phytomeres relies on both the phenology and the appearance model. Shoot length simulations are possible by combining predictions from all of the three developed models. As a general input for model building and simulation local hourly air temperature is required.

## 3. Results and Discussion

### 3.1. Estimated Cardinal Temperatures for Riesling Development

We found optimal parameters to predict growth stages to be $T_{\mathrm{base}}=10.8\;{}^{\circ }C$, $T_{\mathrm{opt}}=19\;{}^{\circ }C$ and $T_{\mathrm{upper}}=24.7\;{}^{\circ }C$. The optimal solution is associated with $\Delta \bar{ODR}=4.9\times 10^{-3}$, $E_{\mathrm{year},\mathrm{phen}}=3.2\times 10^{-2}$ and $E_{\mathrm{year},\mathrm{apex}}=3.9\times 10^{-5}$. The in- and out-of-sample predictive capabilities are ${\mathrm{NRMSE}}_{\mathrm{train},\mathrm{phen}}\approx 0.1518$ ($R^{2}=0.8837$), ${\mathrm{NRMSE}}_{\mathrm{test},\mathrm{phen}}\approx 0.1470$, ${\mathrm{NRMSE}}_{\mathrm{train},\mathrm{apex}}\approx 0.1872$ ($R^{2}=0.8797$), ${\mathrm{NRMSE}}_{\mathrm{test},\mathrm{apex}}\approx 0.2159$. These cardinal temperatures are accepted as the optimum solution that minimizes the difference between $\hat{\mathrm{CDD}}$ and $\hat{R_{\mathrm{apex}}}$ between years with similar organ development rates in both models, while providing an overall good fit to the data.

Results of the first grid search lead to a Pareto optimization considering 306 different cardinal temperature triplets of which $T_{\mathrm{base}}=11{}^{\circ }C$, $T_{\mathrm{opt}}=19{}^{\circ }C$ and $T_{\mathrm{upper}}=25{}^{\circ }C$ was most frequently associated with a Pareto optimum ($n_{\mathrm{opt},\mathrm{iter}1,\max}=67$). The refinement around the optimum from iteration one leads to a subset of 4337 cardinal temperature triplets and, hence, 4031 additional combinations that were subjected to the final Pareto optimization run. Min-max normalization of $\Delta \bar{ODR}$, $E_{\mathrm{year},\mathrm{phen}}$ and $E_{\mathrm{year},\mathrm{apex}}$, within the data subset for the final Pareto optimization was realized with $\min(\Delta \bar{ODR})\approx 3.09\times 10^{-3}$ and $\max(\Delta \bar{ODR})\approx 0.05$; $\min(E_{\mathrm{year},\mathrm{phen}})\approx 6.29\times 10^{-6}$ and $\max(E_{\mathrm{year},\mathrm{phen}})\approx 0.05$; and $\min(E_{\mathrm{year},\mathrm{apex}})\approx 1.07\times 10^{-5}$ and $\max(E_{\mathrm{year},\mathrm{apex}})\approx 0.05$, where the upper limits of approx. 0.05 are a result of the fixed threshold used to extract the data subset.

As expected the refinement did reduce the dominance of a single temperature set as observed in the first iteration ($n_{\mathrm{opt},\mathrm{iter}2,\max}=49$); however, not one of the previously also high rated alternatives was present in the top 10 from iteration two (Table 3).

**Figure 3:**
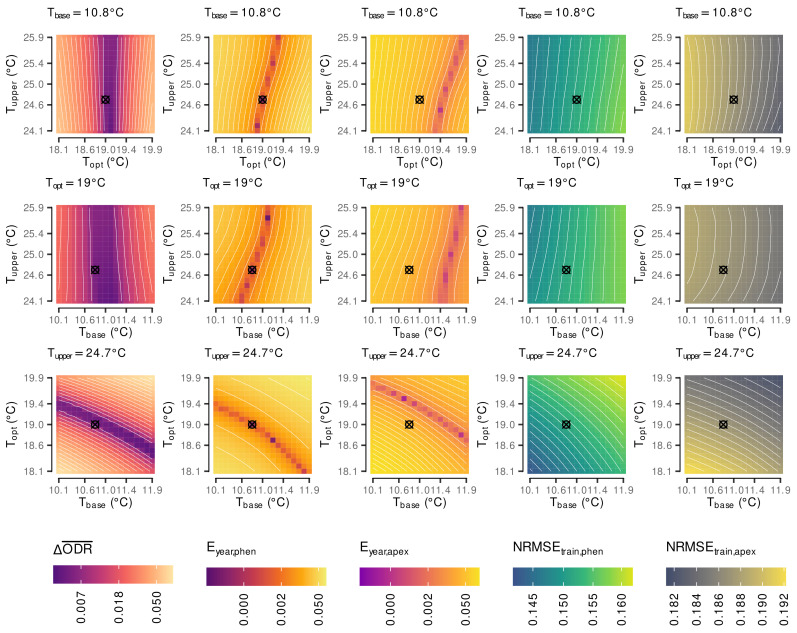
Responses of the five objectives ( $\Delta \bar{ODR}$, $E_{\mathrm{year},\mathrm{phen}}$, $E_{\mathrm{year},\mathrm{apex}}$, ${\mathrm{NRMSE}}_{\mathrm{train},\mathrm{phen}}$, ${\mathrm{NRMSE}}_{\mathrm{train},\mathrm{apex}}$ ) from the refined grid search (step size 0.1$\;{}^{\circ }C$) around the optimum from the first iteration ($T_{\mathrm{base}}=11\;{}^{\circ }C$, $T_{\mathrm{opt}}=19\;{}^{\circ }C$ and $T_{\mathrm{upper}}=25\;{}^{\circ }C$) keeping one cardinal temperature fixed to the estimated overall optimum (⊗).

The refinement around the first iteration’s optimum could further improve the results, but they were generally quite close together (Figure 3). The association with more Pareto optimal solutions of the optimum from iteration 2 compared to the one from iteration 1 might be vastly attributed to the improvement in $E_{\mathrm{year},\mathrm{phen}}$ from 3.0 × 10^−3^ to 3.9 × 10^−5^. The other two objectives, $\Delta \bar{ODR}$ and $E_{\mathrm{year},\mathrm{apex}}$, were less effected. The results from the first, coarse grid search (Figure A2) show that all response variables were sensitive to each of the cardinal temperatures. For example, if $T_{\mathrm{base}}$ is fixed at $11{}^{\circ }C$ while $T_{\mathrm{opt}}$ and $T_{\mathrm{upper}}$ deviate from their optimal values (Figure A2, first line) this has less influence on the NRMSE values, but a significant influence on $\Delta \bar{ODR}$, $E_{\mathrm{year},\mathrm{phen}}$ and $E_{\mathrm{year},\mathrm{apex}}$. Here, the interim optimum lies at the intersection of curve-linear regions with low (good) response values of all three variables. Fixing $T_{\mathrm{opt}}=19\;{}^{\circ }C$ or $T_{\mathrm{upper}}=25\;{}^{\circ }C$ (Figure A2, 2nd/3rd row) shows that ${\mathrm{NRMSE}}_{\mathrm{train},\mathrm{phen}}$ and ${\mathrm{NRMSE}}_{\mathrm{train},\mathrm{apex}}$ react controversial on changes of these two cardinal temperatures. For example, when decreasing both $T_{\mathrm{opt}}$ and $T_{\mathrm{base}}$ ${\mathrm{NRMSE}}_{\mathrm{train},\mathrm{phen}}$ decreases, but ${\mathrm{NRMSE}}_{\mathrm{train},\mathrm{apex}}$ increases. With fixed $T_{\mathrm{opt}}=19{}^{\circ }C$ especially $\Delta \bar{ODR}$ and $E_{\mathrm{year},\mathrm{apex}}$ are more effected by deviations of $T_{\mathrm{base}}$ from its optimum than by changes of $T_{\mathrm{upper}}$. However, fixing $T_{\mathrm{upper}}$ to $25{}^{\circ }C$ $\Delta \bar{ODR}$ and $E_{\mathrm{year},\mathrm{apex}}$ stronger react on deviations of $T_{\mathrm{opt}}$ rather than of $T_{\mathrm{base}}$.

Overall there is a high degree of interaction between all three cardinal temperatures. This can be explained by the high flexibility of the underlying temperature response function (Equation (1)). With the estimated cardinal temperatures the shape of the temperature response function is almost symmetric around $T_{\mathrm{opt}}$ (Figure A3), as the differences between $T_{\mathrm{opt}}$ and $T_{\mathrm{base}}$ and $T_{\mathrm{opt}}$ and $T_{\mathrm{upper}}$ are almost identical. Reducing the gap between the optimum and the upper temperature limit more strongly affects the response function than reducing the gap between optimum and base temperature (Figure A3).

**Figure 4:**
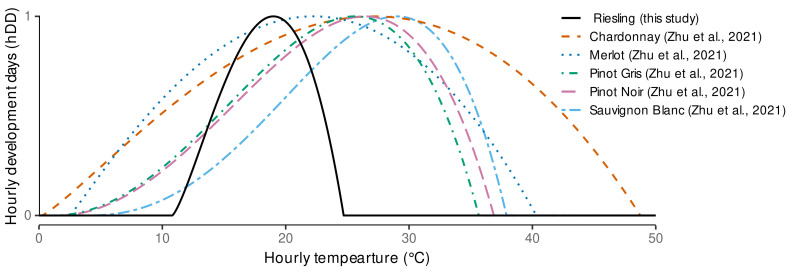
Comparison of shapes of the hourly development days response function (Equation (1)) for different calibrations (this study: ($T_{\mathrm{base}}=10.8\;{}^{\circ }C$, $T_{\mathrm{opt}}=19\;{}^{\circ }C$, $T_{\mathrm{upper}}=24.7\;{}^{\circ }C$)) versus calibrations for five difference varieties from Zhu et al. [28].

The grid search results support the limits we set for the parameter space as none of the optimal temperatures fell on the edge of the search space (Figure A2). However, Zhu et al. [28] found different cardinal temperatures when calibrating different varieties for the duration of two phenological stages later in the season with $T_{\mathrm{base}}$ in the range of 0.2–3.9 ${}^{\circ }C$, $T_{\mathrm{opt}}$ between 22.2 ${}^{\circ }C$ and 29 ${}^{\circ }C$ and $T_{\mathrm{upper}}$ between 35.7 ${}^{\circ }C$ and 48.8 ${}^{\circ }C$. Comparing their hDD-response function shapes to the one estimated in this study (Figure 4), shows that in our case no growth and development is accounted for average hourly temperatures below 10.8 ${}^{\circ }C$ and above 24.7 ${}^{\circ }C$, while the varieties under study in Zhu et al. [28] would strongly respond to higher or lower temperatures. In particular Chardonnay, with its also almost symmetric response function resulting from $T_{\mathrm{base}}=0.239618\;{}^{\circ }C$, $T_{\mathrm{opt}}=27.149\;{}^{\circ }C$ and $T_{\mathrm{upper}}=48.75\;{}^{\circ }C$, seems to be quite insensitive to moderate temperatures. In contrast to this study, Zhu et al. [28] set stronger boundaries on the search space for model calibration (personal communication) with $T_{\mathrm{base}}\in \left[0,5\right]$, $T_{\mathrm{opt}}\in \left[20,35\right]$ and $T_{\mathrm{upper}}\in \left[35,50\right]$. These boundaries do not include our derived optimum cardinal temperature triplet of $T_{\mathrm{base}}=10.8\;{}^{\circ }C$, $T_{\mathrm{opt}}=19\;{}^{\circ }C$ and $T_{\mathrm{upper}}=24.7\;{}^{\circ }C$.

**Figure A1:**
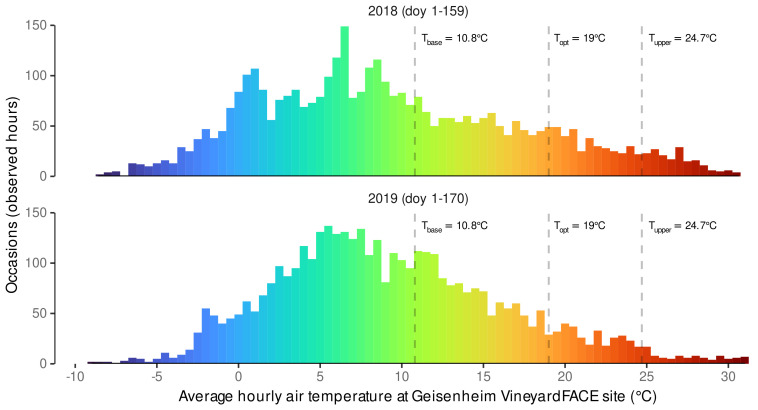
Histogram of average hourly air temperature at Geisenheim VineyardFACE site during the phenological observations. Input data for estimating cardinal temperatures.

**Figure A2:**
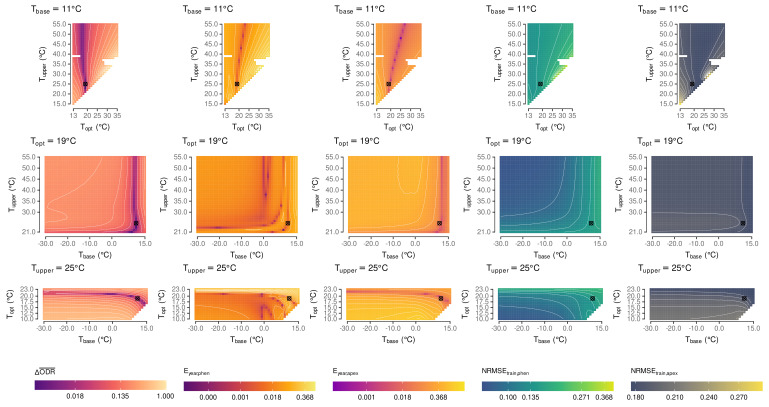
Responses of the five objectives ( $\Delta \bar{ODR}$, $E_{\mathrm{year},\mathrm{phen}}$, $E_{\mathrm{year},\mathrm{apex}}$, ${\mathrm{NRMSE}}_{\mathrm{train},\mathrm{phen}}$, ${\mathrm{NRMSE}}_{\mathrm{train},\mathrm{apex}}$ ) from the coarse, first grid search (step size 1${}^{\circ }C$) keeping one cardinal temperature of the estimated (temporal) optimum (⊗) ($T_{\mathrm{base}}=11{}^{\circ }C$, $T_{\mathrm{opt}}=19{}^{\circ }C$ and $T_{\mathrm{upper}}=25{}^{\circ }C$) fixed.

**Figure A3:**
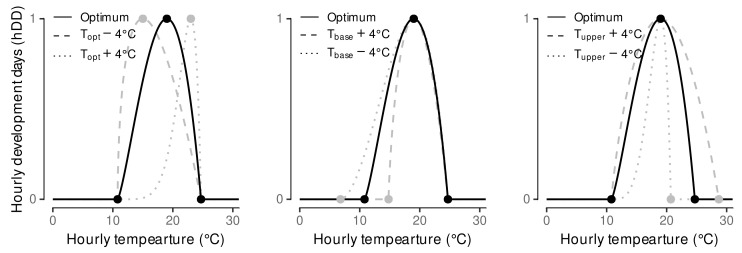
Response of the hourly development days function (Equation (1)) to changes ($\pm 4{}^{\circ }C$) in the optimum parameter set ($T_{\mathrm{base}}=10.8\;{}^{\circ }C$, $T_{\mathrm{opt}}=19\;{}^{\circ }C$, $T_{\mathrm{upper}}=24.7\;{}^{\circ }C$).

The calibration of Cortázar-Atauri et al. [33] to predict phenology stages for seven different grapevine varieties was even more limited by only estimating $T_{\mathrm{opt}}$ with fixed $T_{\mathrm{base}}$ = 0 ${}^{\circ }C$ and $T_{\mathrm{upper}}$ = 40 ${}^{\circ }C$. Hence, with $T_{\mathrm{opt}}$ in the range of 25.6 ${}^{\circ }C$ to 28.5 ${}^{\circ }C$ their results are more similar to to the calibration from Zhu et al. [28] than to our estimates. A direct comparison with other methods also estimating temperature triplets might be problematic, due to different response functions. Nevertheless, Molitor et al. [117] found a temperature triplet of 7 ${}^{\circ }C$, 18 ${}^{\circ }C$ and 24 ${}^{\circ }C$, more close to our estimates. They applied the model from Molitor et al. [19] which uses linear response functions between the threshold temperatures on Riesling data from three different locations using daily mean temperatures as input data. Here the highest temperature represents a heat threshold above which the response value starts to decrease. The upper limit associated with a zero-response can be estimated to 35 ${}^{\circ }C$ using a function depending on the temperature triplet [19]. The calibration procedure from Molitor et al. [19] is also very restrictive on possible temperatures by limiting the lower threshold to integers from 3 to 7 ${}^{\circ }C$, upper thresholds between 15 to 21 ${}^{\circ }C$ and heat thresholds from 21 to 25 ${}^{\circ }C$. If we focus only on our estimated base temperature of $T_{\mathrm{base}}$ = 11.8 ${}^{\circ }C$, it lies in close proximity to the long-term base temperature of 10 ${}^{\circ }C$ used in the grapevine modeling [42,118,119,120]. We did not observe local average hourly air temperatures above 31 ${}^{\circ }C$ or below −9 ${}^{\circ }C$ (Figure A1) within the considered time frame in each year starting from 1 January. However, this should not have prohibited estimating cardinal temperatures outside this range, as the shape of the response function is highly flexible (Figure 4 and Figure A3). Our new approach is an advancement to the models used by Schmidt et al. [12] as now a non-linear response function depending on the sub-daily temperature course relying on hourly time steps is introduced. The use of hourly temperatures is generally recommended to reduce approximation errors compared to daily measurements, while only negligible accuracy improvements are obtained when further reducing the time step [121]. While the CDD model depends on three cardinal temperatures that have a clear biological meaning in other studies, the calibration was still carried out according to purely statistical concepts, so the estimated temperatures should not be misused for other purposes. Hence, it follows, for example, that the upper threshold temperature estimate of 24.7 ${}^{\circ }C$ must be seen in the context of the measured data. We rely on hourly temperature measurements to model observations with an observation frequency of days or weeks. Thus, if the statistically optimal solution can be achieved with higher weighting of lower temperatures, consequently, more extreme temperatures are associated with less or no influence. In regions where Riesling is grown, even during the course of a very warm day with peak temperatures well above $T_{\mathrm{upper}}$, there will be enough hours with temperatures below $T_{\mathrm{upper}}$ that the model will be able to account for growth on that day. This aspect also applies to phenological models discussed above and shows that comparability of such estimated temperature thresholds is limited. However, this does not contradict the fact that models developed are valid empirical models.

With combining phenological ratings and digitization data in the calibration we aimed at more robust estimates of cardinal temperatures. Furthermore, the same CDD estimation is now used for the growth and development models, i.e., used in the following to predict phenological stages, in particular budburst, and shoot growth, thus reducing the overall parameter count of a FSP model.

### 3.2. Phenology Prediction

#### 3.2.1. Model Reduction

Parameter estimation using grid search optimization required a vast amount of model fits; hence, it was conducted using a non-Bayesian model to be computational feasible. With optimal parameter set, the model was transferred to an equivalent Bayesian model that was then subjected to a step-wise model reduction to predict the CDD for observed growth stages, especially budburst (linearized E-L-system stage no. 4). Based on estimated performance measures, especially LOOIC, it was found that predictive capabilities did not suffer from removing all fixed effects except ${\mathrm{ELSt}}_{\mathrm{linear}}$ (Table 4).

Only the intercept and the ${\mathrm{ELSt}}_{\mathrm{linear}}$ were associated with a pd of 100%, which further justifies the conducted model reduction. It is not surprising that we could not estimate any year-effect, as this has been one initial assumption in the estimation of the cardinal temperatures. Nevertheless, the transported information were consistent across the step-wise process, and hence we can also conclude that no substantial effect of ambient and elevated CO${}_{2}$ conditions on necessary cumulative development days could be estimated for the considered growth stages. This stands in contrast to the results from Lüscher et al. [122] showing an effect of extreme CO${}_{2}$ enrichment (700 ppm) on duration of a phenological phase. An enrichment to only approximately 480 ppm (aCO${}_{2}$ + 20%) [88] might not be enough to induce the same effects. In addition, we also refitted frequentist versions of the final and the full model that were compared using AICc, a second-order Akaike Information Criterion [96]. Comparing the refitted frequentist versions, both using the full data set, the final model had a lower AICc indicating a better performance than the full model with values of 12,139.95 and 12,150.75, respectively. In both cases, removing the random slope did worsen AICc values; hence, this was not considered in the Bayesian model selection process.

The final reduced Bayesian model refitted as a generalized linear mixed model by using an exGaussian likelihood substantially improved the model fit based on a considerably lower LOOIC-estimate (Table 4). This might be related to the phenological ratings usually including some shoots further developed than the average shoot, hence, matching the characteristic positive skew of the exGaussian distribution. Unfortunately, this distribution was not available in the frequentist mixed modeling framework and could therefore not already be used in the upstream cardinal temperature estimation procedure.

**Figure 5:**
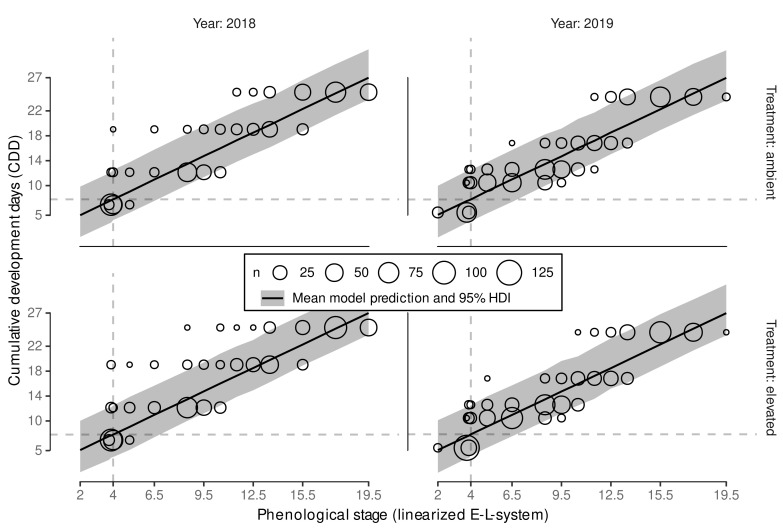
Posterior predictions of the final model fit (no year and treatment effect) using the full data set (final, exGaussian, full data, see Table 4) compared to phenological ratings split by year and treatment over thermal time. Bubbles (∘) show the frequencies (*n*) of observed phenological stages in the linearlized E-L-system.

Lastly, the final Bayesian exGaussian model was updated using the full data set to incorporate all available information (Table 4) with similar parameter values and performance measures (RMSE, R2). Here, the estimated LOOIC can not be compared to the models fitted using the test data only. Using the exGaussian-likelihood did reduce the left tail from posterior predictions, which is especially relevant for predicting necessary CDD for budburst, where it is generally assumed that a certain threshold of heating units needs to be exceeded to initiate budburst. This aspect might have been of even more relevance, e.g., when the starting date for CDD summation would have been chosen to be later in the year reducing the possible total accumulated CDD [25]. Hence, we expect the selected exGauassian-model to be the more robust approach when aiming to predict budburst and its variability. Plotting the predictions against the data (Figure 5) we can see the effect of the exGaussian-model on the the prediction interval that is slightly wider above the model mean than below.

However, the model still relies on our a priori assumptions when linearizing the modified E-L-system stages before flowering to model a linear relation between CDD and ELSt${}_{\mathrm{linear}}$. However, this was necessary to use all ranking data instead of only fitting a model to predict one specific stage (e.g., [28]). With an overall good model performance (see Table 4 and Figure 5) we conclude that our assumptions were reasonable.

**Figure 6:**
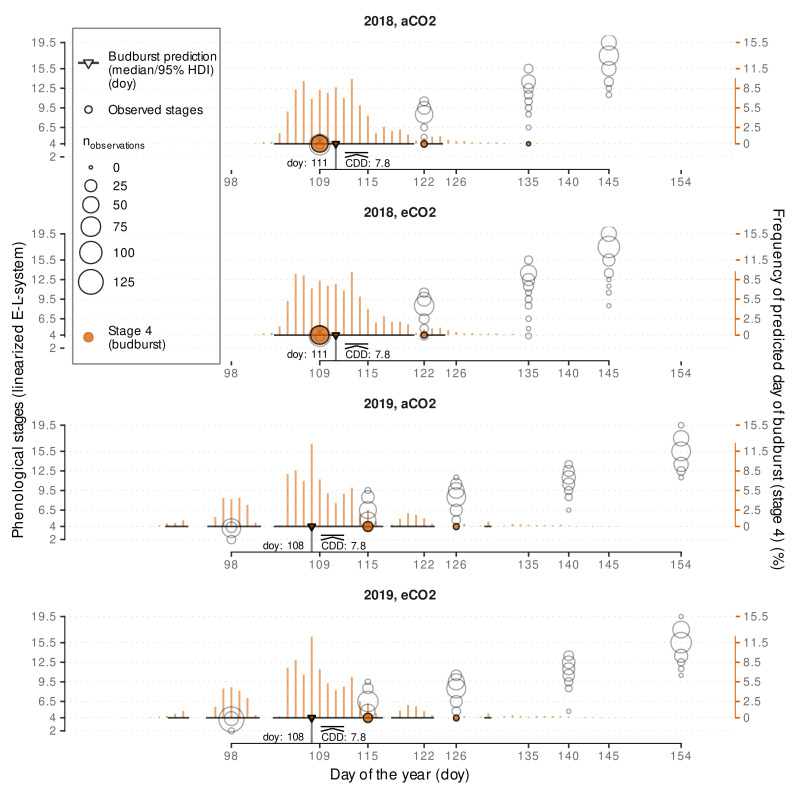
Posterior predictions of budburst date by matching predicted $\hat{\mathrm{CDD}}$ to day of the year (doy). Model data is presented as the median plus the 95% highest density interval (—▼—) and as frequencies of predicted budburst for each doy (%) with $n_{sim}=8000$ per sub-plot (vertical orange lines). While the model does not differentiate between years and treatment, predictions can differ because of random sampling from the posterior distribution. The data (∘) includes all phenological ratings in the linearlized E-L-system, not only budburst, where the bubble size indicates the respective count ($n_{\mathrm{observations}}$).

A dynamic functional-structural plant model requires an accurate estimation of budburst date. The Bayesian model calibration allows predicting several possible predictions of CDD ($\hat{\mathrm{CDD}}$) using posterior predictions. Aligning the $\hat{\mathrm{CDD}}$ with observed CDD of a season, each $\hat{\mathrm{CDD}}$ can be associated with a day of the year. The median predicted $\hat{\mathrm{CDD}}$ for budburst was estimated to $\bar{\hat{\mathrm{CDD}}}\approx 7.8$ (Figure 6) with slight variation due to simulation uncertainties.

It was independent of year and treatment, as inclusion of these effects did not provide any improvements in the model predictive performance and is associated with doy 111 and doy 108 for the calibration years 2018 and 2019, respectively. According to https://rebschutz.hs-geisenheim.de/rebentwicklung/rebphaenologie.php (accessed on 7 February 2022) which relies on the model of Molitor et al. [19], budburst around Geisenheim was projected to doy 109 (19th of April) for both 2018 and 2019.

By using a Bayesian approach we can now estimate a budburst doy for single shoots, hence, incorporating natural observed budburst variability into the plant growth model. In Figure 6 we show the relative frequency and the 95% highest density interval (HDI) based on 8000 posterior predictions of the CDD for ${\mathrm{ELSt}}_{\mathrm{linear}}=4$ per variant. Highest density intervals in 2018 and 2019 cover a range of approx. 20 days and 40 days, respectively, but include gaps that can be related to cold periods where no additional CDD accumulate, and hence these dates are not associated with budburst events when matching the predicted CDD threshold to the first date of accumulation. Since, in contrast to the quantiles, the highest density interval captures the most probable observations, these gaps become visible. The high variability of predicted budburst days coincides well with the stretch in the observations of budburst.

#### 3.2.2. External Validation of Budburst Data Predictions

**Figure 7:**
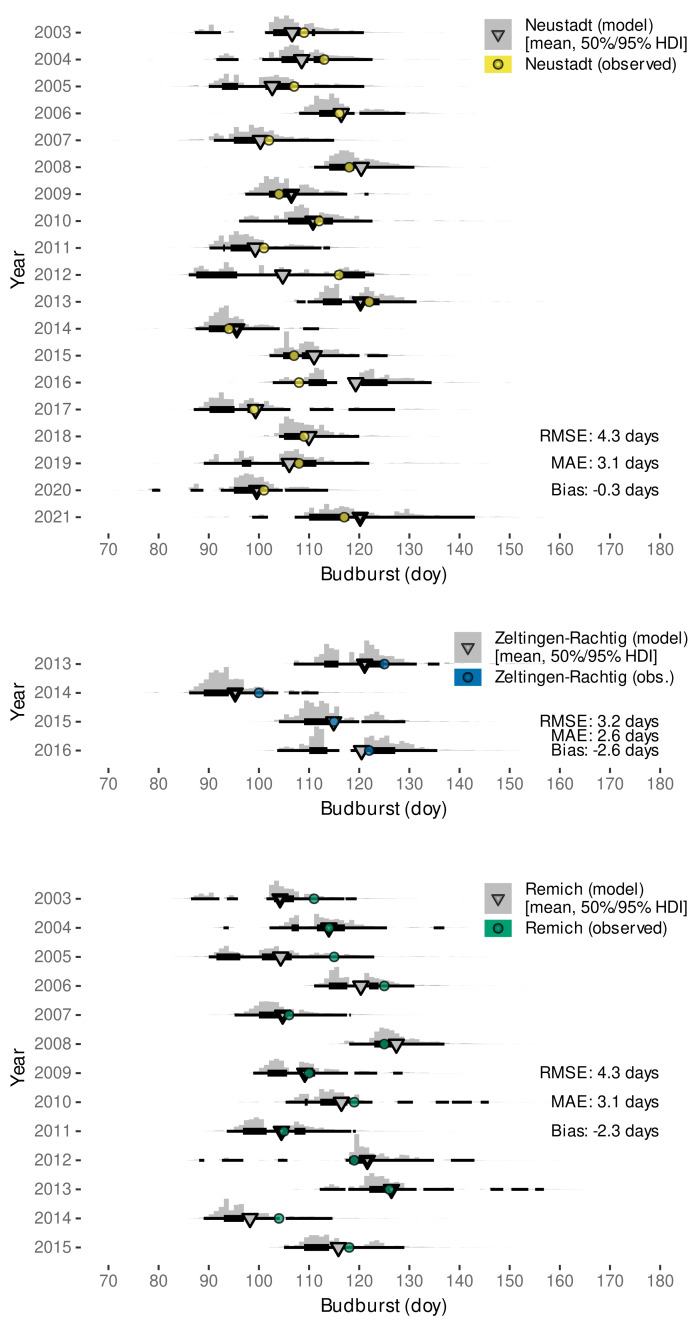
Posterior predictions of budburst date by matching predicted $\hat{\mathrm{CDD}}$ to day of the year (doy). Model data is presented as the mean and the ranges between 50% and 95% highest density intervals (HDI) (—▼—). Observations (•) include data on Riesling vines monitored in Neustadt an der Weinstraße, Germany, in Zeltingen-Rachtig, Germany, and Remich, Luxembourg. Gaps in range bars are a result of the highest density interval estimation and related to the temperature course within a year.

Validating the predictive capabilities of the final Bayesian model with external data sets, we found root-mean-squared errors between 3.2 to 4.3 days depending on the location (Figure 7).

On all occasions, the observed date fell within the estimated range of budburst (95% HDI), often even within the 50% HDI. Only the data set from Neustadt included the years 2018 and 2019 that were used for parametrization, suggesting that the Bayesian model calibration only using phenology ratings from one site and two subsequent seasons already yields robust predictions for Riesling grown at cool-moderate climate regions in different years. Estimated bias might not only be related to model calibration specifics but could also be a consequence of e.g., vineyard microclimate, which might differ from the local weather station data [21,123]. In addition and similar to other studies [19], observed bias might also be related to different clone-rootstock combinations or subjective interpretation of the phenological growth stages.

The model from Schmidt et al. [12] had an overall similar performance for Neustadt, Zeltlingen-Rachtig and Remich with RMSE of 4.1, 4.0, 3.2 days and bias of 0.5, −3.1, −2.0 days, respectively. However, the variability in the approach from Schmidt et al. [12] lead to some outliers where budburst would be predicted to days before the 1 January of the respective year due to the random sampling from a normal distribution around the estimated budburst date. This problem was not found for the novel model which is based on an exGaussian distribution. Nevertheless, expecting warming winter temperatures in the future, budburst variability in Europe might further stretch towards the beginning of the year. For example, in southern Italy a budburst event has already been observed on 11 January 2020 [124]. While the novel model is based on both ambient and elevated data, we could not yet reliably distinguish between aCO${}_{2}$- and eCO${}_{2}$-grown plants. However, it is not excluded that future data might allow a differentiation that could further improve predictions for historic (aCO${}_{2}$) and/or future (eCO${}_{2}$) conditions.

**Figure 8:**
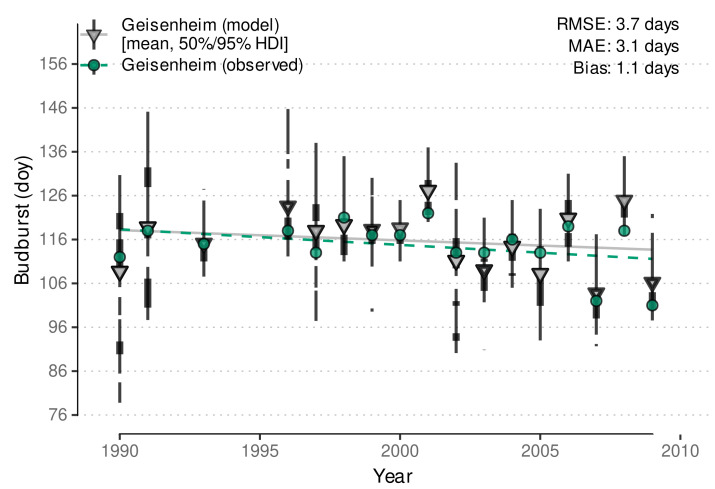
Observed advancement of budburst captured by posterior predictions of budburst date by matching predicted $\hat{\mathrm{CDD}}$ to day of the year (doy). Model data is presented as the mean and the ranges between 50% and 95% highest density intervals (HDI) (—▼—). Observations (•) include data on Riesling vines monitored in Geisenheim, Germany [14]. Regression lines for model and observation are based on the means.

Stoll et al. [14] noted an advancement of phenological stages in the Rheingau wine region (Germany) as an effect of climate change. Our model successfully recovers the advancement of budburst observed between 1990 and 2010 in the model predictions (Figure 8). Again, predictions transport high variability of budburst dates within a year, but a similar trend is detected. The low RMSE (3.7 days) and the low bias (1.1 days) confirm the previously determined good accuracy of the model predictions. This suggests the developed model is capable to be used for climate impact studies relying on budburst predictions. The model from Schmidt et al. [12] yielded similar RMSE (3.4 days) and bias (0.3 days) for this data set, but with the same outlier problem as stated above.

#### 3.2.3. External Validation by Projections of Beginning of Flowering Date

**Figure 9:**
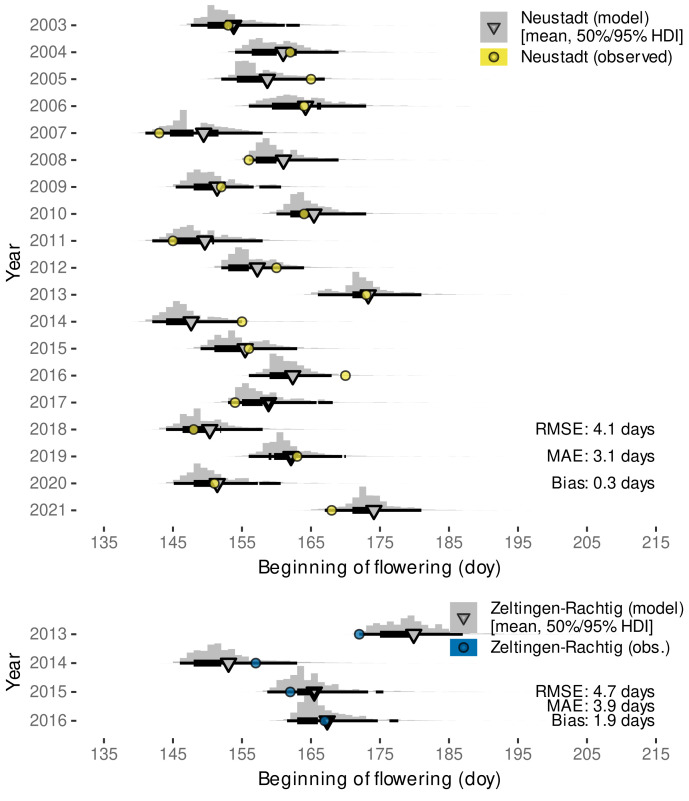
Posterior predictions of beginning of flowering (about 16 leaves seperated; linearized E-L stage 21.5) date by matching predicted $\hat{\mathrm{CDD}}$ to day of the year (doy). Model data is presented as the mean and the ranges between 50% and 95% highest density intervals (HDI) (—▼—). Observations (•) include data on Riesling vines monitored in Neustadt an der Weinstraße, Germany, and in Zeltingen-Rachtig, Germany. Gaps in range bars are a result of the highest density interval estimation and related to the temperature course within a year.

The external data sets from Zeltlingen-Rachtig and Neustadt also include observations on beginning of flowering date. As this stage is directly following the stages considered in the calibration, we chose to project this adjacent stage with our model as ELst${}_{\mathrm{linear}}=21.5$ by extending the linearized E-L-system. With RMSE-values of 4.1 days and 4.7 days these posterior predicted projections agree similarly well with the observations (Figure 9) as the budburst predictions. The majority of observations fell into the estimated 95% HDI that covers approximately 10–15 days. However, the bias for both locations changed sign, albeit at a generally low level, indicating that the forecasts for beginning of flowering are on average somewhat later in the year than the corresponding observations. This might be a consequence of the simple extrapolation of the linearized E-L-system, despite the beginning of flowering stage being only partially related to the number of leaves (‘about 16’), and mainly characterized by ‘first flower caps loosening’ [35]. While not yet considered in the functional-structural plant model on Riesling vine [12], these results suggest that one might use the developed model to also predict beginning of flowering that could become relevant when berry development is included in future versions [46,125]. Furthermore, due to the synchronization of organ development rates in the estimation of cardinal temperatures, we expect a good agreement with the speed of the architectural development of the plants between budburst and the beginning of flowering.

### 3.3. Primary Shoot Internode Appearance

#### 3.3.1. Model Reduction

Primary shoot growth was split into a model for internode appearance and a model for internode growth. We already assumed a linear dependency between the rank of the apex and cumulative development days up to the maximum observed apex rank of 23 when estimating the cardinal temperature triplet using a frequentist linear mixed model.

In the following, Bayesian model calibration was used and the model selection process evaluated the effects of the additional factors year and treatment (aCO${}_{2}$, eCO${}_{2}$) including the interaction of CDD×trt (Equation (6)). The group-level effects model structure was fixed to control for the sampling structure given the experimental design (cf. Equation (6)). It was found that model performance was not affected by first removing the interaction and then also both factors year and treatment entirely. We also tested whether including year as a group-level effect (GE) would be an alternative, but this was not the case. Hence, the selected final model, although not with the overall lowest LOOIC, but with a negligible difference to more complex models, does not include any year effect and predicts best the apex rank only considering CDD. Fulfilling the initial assumption of no year effect is in accordance with the results from Schultz [42] on plastochron development rates. The model selection progress included a training and test data set, while the hold-out-data RMSE confirmed the overall good model performances by being in line with the training data RMSE (Table 5). The choice of the final model was supported by the fact that the additional parameters (trt, year) were only associated with pd-values of <72%, i.e., not indicating a significant effect. Following the model selection progress, the final model formula was refitted using the full data set to incorporate as much information as possible in the Bayesian calibration, i.e., to estimate the slope and its variability, as this information is supposed to be used as an input in functional-structural plant modeling.

Final model results estimated an average slope ($\bar{IAR}$) of 0.7784 ranks/CDD with a standard deviation of 0.081 ranks/CDD when including the estimated uncertainty of the random slopes per shoot. The reciprocal $1/\bar{IAR}$ of 1.285 CDD/rank indicates how many CDD are necessary for a new internode to appear. This is in accordance with the estimated appearance rate (slope) of the Bayesian phenology model using the Gaussian likelihood with a value of 1.289, and reflects the assumptions we made in the cardinal temperature optimization. The estimated slope in the subsequently established exGaussian phenology model is only slightly lower (1.2577) (Table 4), and therefore not to be seen as a contradiction.

**Figure 10:**
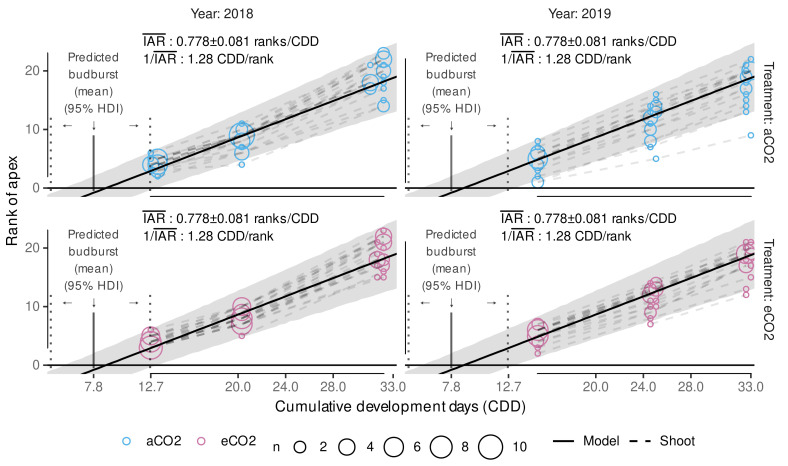
Model fit of maximum ranks (apex rank) over cumulative development days (CDD) (mean (–) and posterior predicted quantile interval (Q2.5–Q97.5); gray area) versus data split by the two different years (2018, 2019) and the two treatments (aCO2, eCO2). Bubbles (∘) show the frequencies (*n*) of observation data. Model data is equal in all panels, as no treatment or year effect was estimable. Dashed lines connect data from the same shoot. Predicted budburst CDD from the phenology model is indicated, too (mean and 95% highest density interval).

The intersection of the model regression line with the estimated CDD for budburst approximately coincides with an apex rank of 0 (Figure 10). While the focus of this model was the estimation of the slope parameter, this supports the validity and assumptions of the two analyses, the estimation of the budburst CDD and this analysis on phytomer development. The slight offset might be related to the fact that the observational digitization date of the apex rank does not perfectly coincide with the appearance of that rank. We expect a negative offset, i.e., the appearance should have happened a little earlier. However, since only the slope will be used in an FSP model, this aspect is only considered in the internode growth model, where we try to control for this uncertainty (see Section 2.8). Thus, it has been shown that the estimated cardinal temperatures for the CDD calculation allow predicting phenological stages and phytomer development rate at the same time. The mixed modeling approach, where the development of each single shoot is considered in the mixed model part, was necessary to give a robust estimate of the average development rate and its variability, as the phenology ratings and model results already indicated that there is between-shoot variability in the development, i.e., the shoots were never all at the same stage of development. To account for this, the modeling structure controlled for individual differences in the intercept and slopes. A consequence of individual slopes and intercepts can be seen in the model prediction interval that slightly increases with increasing CDD (Figure 10).

For the transfer of these findings into a stochastic plant growth model we are only interested in the estimated development rate and its variability, as the onset of development should be estimated using the phenological model and its estimated uncertainties for budburst CDD. As stated above, combining the posterior distribution of the slope’s fixed effect with random samples from the slope’s group-level effect (modeled as a Gaussian distribution) we arrive at a posterior distribution also following a Gaussian distribution with N∼(μ=0.778 ranks/CDD, σ=0.08 ranks/CDD). With no additional effect in the model we can rely on just this single sampling distribution to assign a development rate to each single shoot within a stochastic FSP model.

#### 3.3.2. External Validation of Appearance Rate

**Figure 11:**
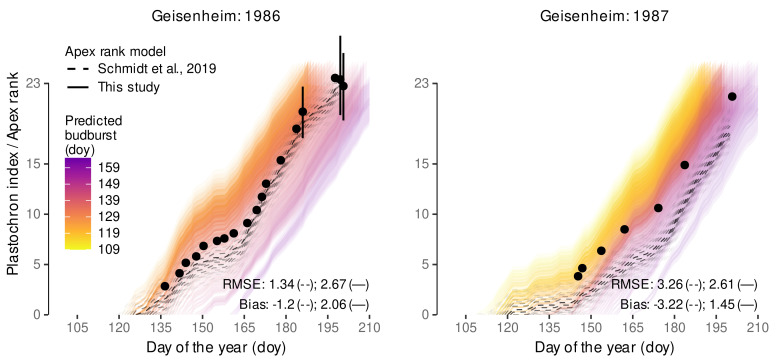
Model predictions (one line per predicted budburst doy) compared to observation data on plastochron index (=node rank; •) from Schultz (Figure 8 (‘S-System’) [42]) Riesling vines grown 1986 and 1987 at Geisenheim, Germany, using the model developed in this study (colored solid lines) and the one from Schmidt et al. [12] (dashed lines). Shaded areas around the predictions from this study represent uncertainty associated with the estimated variability in the internode appearance rate ($\bar{\mathrm{IAR}}\pm 1\times \mathrm{SD}$). Measurement data was extracted from the original figure using Webplotdigitizer [114]. Where extractable the error interval describes ±2×SE (**|**). Predictions are limited to the calibration range with a maximum observed apex rank of 23.

The validation results show good agreement with measurement data within the calibration time frame (max. apex rank = 23) (Figure 11). While the one-time best predictive performance is associated with the model from Schmidt et al. [12] (dashed lines) regarding the 1986 data, the novel model yields more consistent results in both years. This is in line with the aim to develop a more robust model, which might not always yield best point-wise predictions. Beyond this, Schultz [42] found a decreases in plastochron development rate with advancing thermal time, suggesting the linear trend might overpredict data currently out of scope. Yet, with additional data, the model could be adapted to a limited growth model, where the appearance rate decreases with CDD in the later season. However, quantifying the decrease of organ appearance rate with thermal time would require the absence of standard management practices in a vineyard, in particular shoot trimming, or the effects of timing and intensity of shoot trimming on shoot development need to be taken in account in a model, too.

### 3.4. Internode Length Model

#### 3.4.1. Model Selection

Model selection was split between frequentist and Bayesian models. First, frequentist non-linear mixed models were fitted to evaluate whether the inclusion of a fixed effect for treatment and/or allowing for heteroscedasticity, i.e., different variances with increasing mean or per rank, is beneficial. Results indicated that especially modeling heteroscedasticity improved model performance regarding AICc (Table A3).

As differences between the inclusion and exclusion of a treatment effect were small, this parameter was also tested in the even more complex Bayesian calibration. The extensions included the switch to a generalized non-linear mixed model, relying on Gamma-regression to restrict predictions to only positive values of internode length (see Section 2.8) and implies the variance to increase with the mean. Heteroscedasticity, i.e., a variability of the shape parameter of the Gamma distribution likelihood, was modeled by group-level effects related to the multilevel sampling structure plus the rank, as this has already proven beneficial in the frequentist pre-analysis. As we were not interested in inferences on these parameters, but wanted to control for their variability, we did not consider any fixed effects here, but we also incorporated the treatment fixed effect on the non-linear parameters, not to overlook anything in the Bayesian approach that might not have been extractable within the pre-analysis due to the less complex model structure.

Within the Bayesian framework, the lowest LOOIC is estimated for the model with treatment effect (Table 6); however, differences in LOOIC are small. Differences of ELPD between the models in Table 6 are all below 4, indicating negligible differences [126]. Hence, we expect no beneficial effect on prediction accuracy when incorporating a fixed treatment or year effect. Thus, we selected the least complex model of all four as the final model—the model where no year effect is considered in fixed and group-level effects. This decision was supported by lower RMSE values for the ‘no year’-model. However, as literature suspects an effect of eCO${}_{2}$ on shoot growth [88] and given the still sparse data, we investigate a possible treatment effect on the outcome in the following.

**Figure 12:**
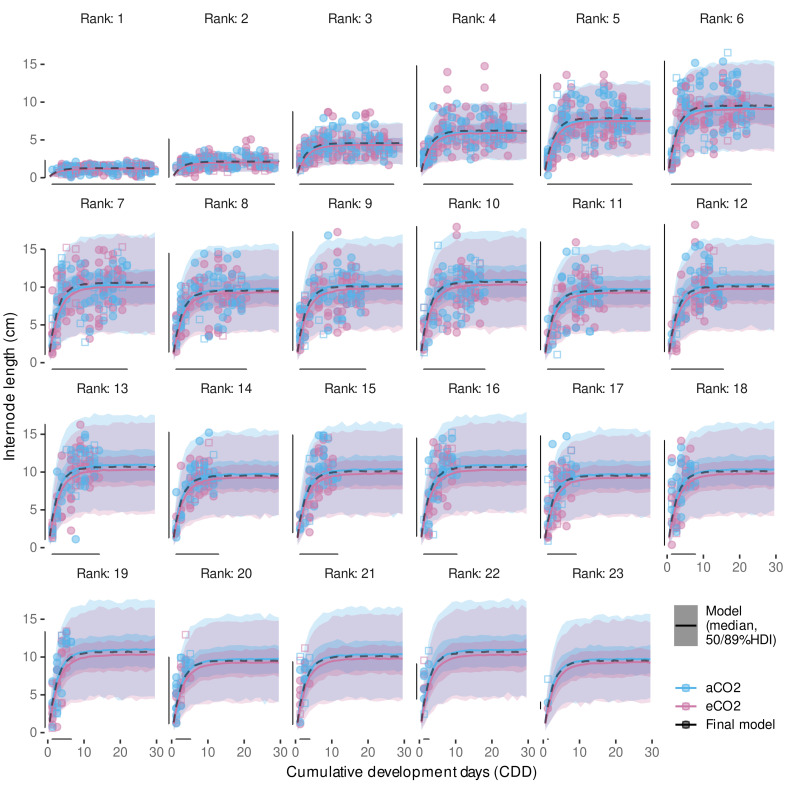
Model predictions (median (–) and the 50% and 89% highest density intervals (shaded area) of internode length considering fixed treatment effect on non-linear model coefficient. The dashed line includes the median prediction of the final model formulation (see Table 6). Data points represent the calibration data set (training; ∘) and the hold-out data (test; □).

**Figure A5:**
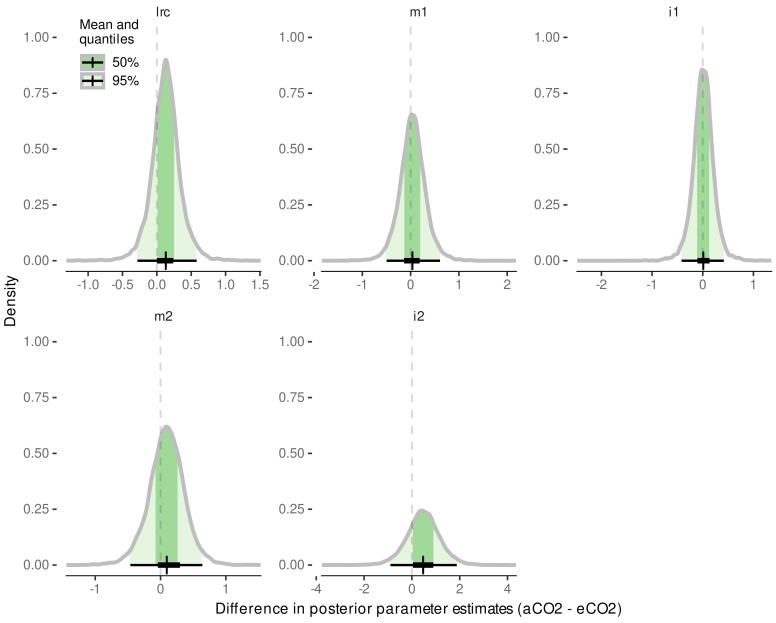
Difference between treatment parameter estimates from the posterior samples of the non-linear model coefficients.

For example, we calculated the difference in parameter estimates between treatments from the posterior samples of the non-linear model coefficients for the fixed treatment effect. Only when considering just the 50% quantile interval we found $i_{2}$-differences to be different from zero (Figure A5). This further indicates that there is no significant difference between eCO${}_{2}$ and aCO${}_{2}$ treatment in modeling internode growth depending on CDD. The data shows substantial variability within parameter estimates, which makes it difficult to detect small differences. However, model predictions with the ‘fixed trt’-effect model while excluding group-level effects further verified that, based on these models, there is also no visible impact when the interplay of the estimated parameters is considered (Figure 12).

**Figure A6:**
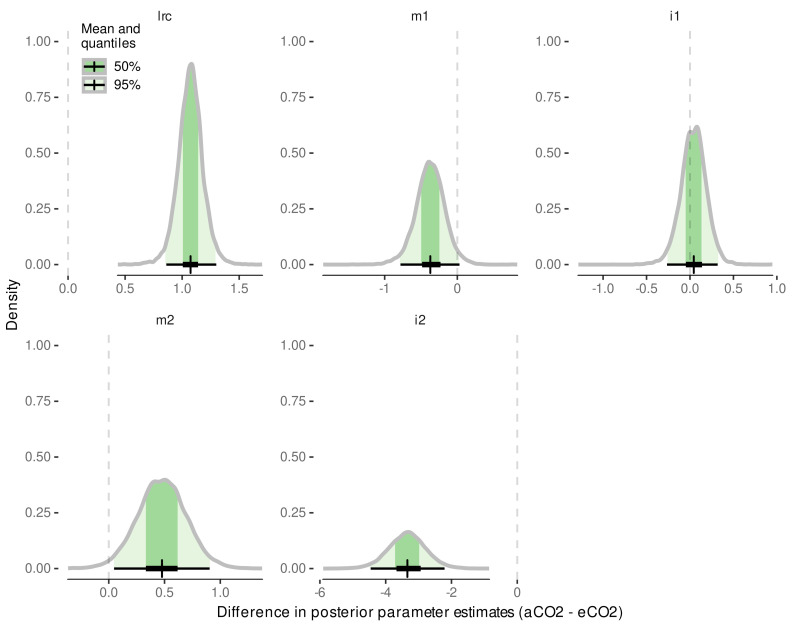
Difference between year parameter estimates from the posterior samples of the non-linear model coefficients.

**Figure A7:**
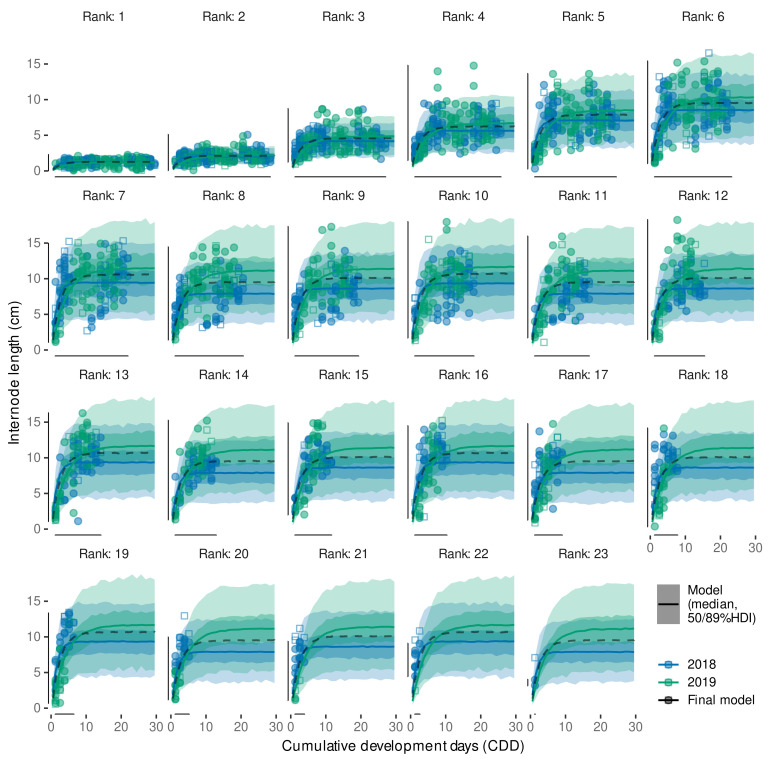
Model predictions (median (–) and the 50% and 89% highest density intervals (shaded area)) of internode length considering fixed year effect on non-linear model coefficient. The dashed line includes the median prediction of the final model formulation (see Table 6). Data points represent the calibration data set (training; ∘) and the hold-out data (test; □).

Similar to the evaluation of how the ‘fixed trt’-effect model performs on predictions, we also investigated the ‘fixed year’-model. In this case, the estimates of the model coefficients between years more often differ substantially from zero (Figure A6). This is transferred into predictions by faster growth in 2018 compared to 2019, related to the difference in the lrc-coefficient (Figure A7). Especially the longer growth duration estimated for the 2019 data could be seen as contradictory to observations from Schultz and Matthews [98], where internode growth was found to last approx. 12 days and was limited to the upper eight internodes. Considering the model estimated IAR, eight internodes would develop within the next 10-11 CDD, which is more in line with the final model’s growth speed, where 99% of the maximum IL is reached after approx. 10 CDD, than with both estimates for the different years (2018: 6 CDD / 2019: 18 CDD until 99% max. IL; cf. Figure A7). However, we also cannot fully rule out some operator bias in the digitization procedure between both seasons that could affect the lrc parameter. Following Schmidt et al. [12] we assumed growth speed (i.e., the lrc-coefficient) to be constant across ranks, while for example Greer and Weston [127] found an increase in elongation duration for internodes along the shoot studying *Vitis vinifera* cv. Semillon grapevines in growth chamber experiments. In addition, the estimated coefficients associate the year 2019 with higher asymptote values, especially for the R>7, mostly related to the differences in $i_{2}$ (Table 6, Figure A6). This might be related to more frequent extreme internode length in the measurements, influencing the average growth curve. Given this contradictory observations and the fact that the model without considering a year effect even performed better based on RMSE measures, the ‘fixed year’-model has not been used any further. If we would have found strong evidence that an effect of the year would lead to a significantly better model, further analysis on underlying effects could have been conducted. However, future work might focus on between year differences of environmental factors, such as (temporal) water stress [98,128], temperature course before budburst [129] or light conditions [53], to refine the model.

**Figure 13:**
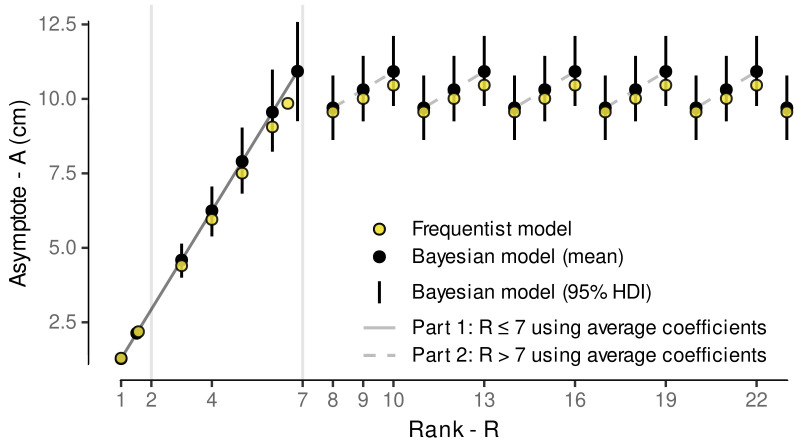
Model parameters for asymptotic values (A) per rank (R) based on training data models fits of the selected Bayesian and frequentist models. Part 1: Rank ≤7; Part 2: Rank >7 (Equation (21)).

The model performance output with no fixed effects indicated another possible model reduction, with the probability of direction for the parameter $m_{2}$ being close to 97.5%. Hence, a subsequent test on removing this parameter from the model was conducted. If $m_{2}$ had proven to be obsolete, this would have indicated a constant asymptotic internode length for ranks > 7, but the model performance was worse (LOOIC 6801.39 [100.88]). Thus, this test did not justify any further model reduction. The final model supports the findings from Schmidt et al. (Figure 4 [12]), where the maximum internode length, i.e., asymptotic values, of internodes at higher ranks (>7) show a repetitive pattern (Figure 13). This could be related to observations from Schultz and Matthews [98] on three morphological distinct internodes related to tendril presence and adjacency. Louarn et al. [99] found irregularities from the normal pattern of succession of such phytomer types in the cultivars ‘Grenache N’ and ‘Syrah’, which would explain, why the posterior distribution for $m_{2}$, the parameter representing the assumed strong systematic behavior, also includes the zero. Comparing the estimated ranges of the asymptotic values for the different ranks based on the Bayesian model, generally lower variability was estimated for higher ranks, but variability also increased with rank for ranks ≤ 7 (Figure 13). As the average parameter estimates from final frequentists and Bayesian model formulations were similar (cf. Table 6 and Table A3), average asymptotic values between both methods were close, too. The modeling choice to incorporate a model formula for the asymptote depending on the rank does reduce model complexity and might allow projections for ranks not yet observed (R>23), but comes with the price that individual per-rank predictions might be less precise, when the assumptions regarding the sub-model are not fully met. Here, we see benefits from using a Bayesian approach, as the posterior distribution also captures the deviations from the estimate’s average for each rank that can be transferred into plant growth simulations.

In the final model the probability of direction for the parameter $s_{R7}$ fell below 99.0%; hence, here also a subsequent test on removing this parameter from the model was conducted. With LOOIC 6745.97 [101.88], this model did not prove to be superior; therefore, $s_{R7}$ has been kept in the model for the final fit to the full data set. This means that the two auxiliary parameters, $s_{R2}$ and $s_{R7}$ have proven to be beneficial for the model. This supports the initial observation that there might be a slight non-linearity towards a Gompertz-like function shape in the dependency of the asymptote on ranks for R<7.

Not all parameters associated with lower pd, e.g., lrc or $m_{1,\mathrm{aCO}2}$, were reviewed in detail as for example lrc overlapping 0 does not indicate ‘no effect’, and aCO${}_{2}$ and year fixed effects models were set-up to provide a parameter value for each variant. Hence, whether these factors do have an effect must be evaluated based on the difference between the parameter pairs (cf. Figure A5 and Figure A6).

#### 3.4.2. Model Predictive Performance

**Figure 14:**
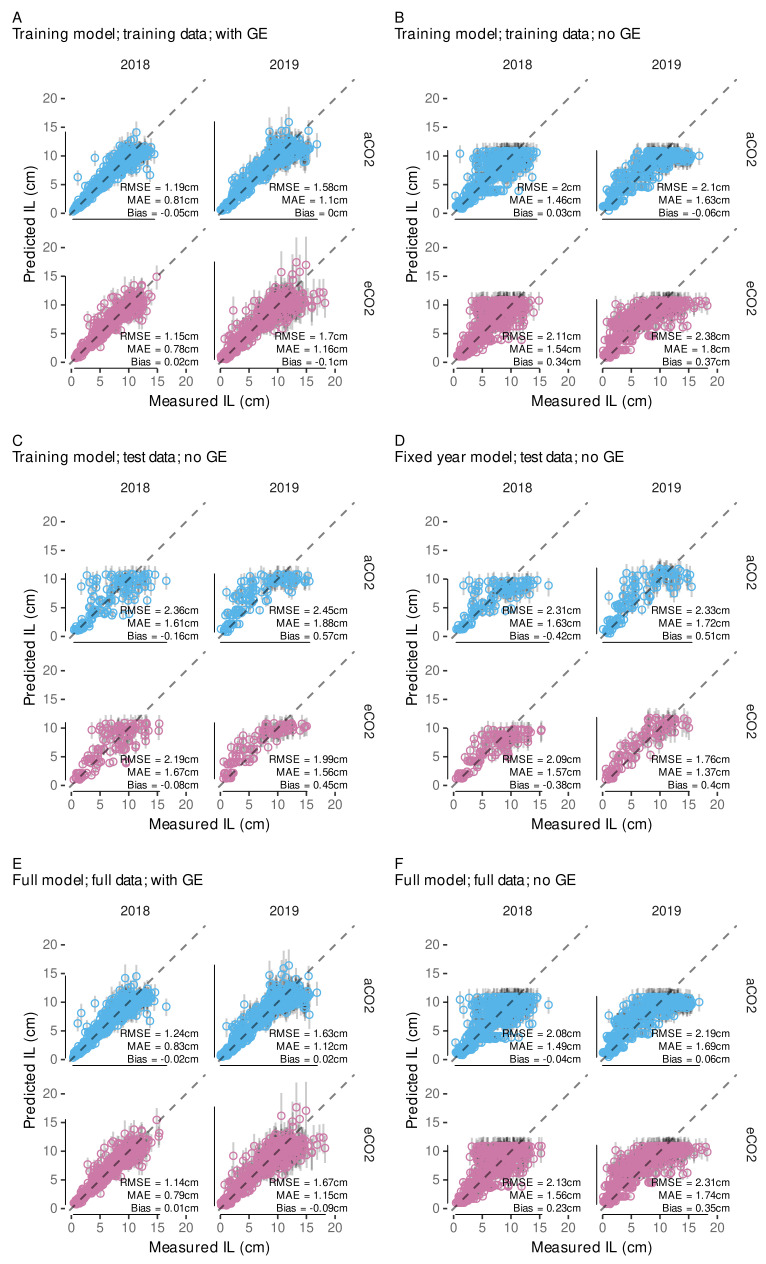
Different model predictions of internode length (IL; cm; mean and 25–75% quantile interval) versus measurement data split by year and treatment (aCO${}_{2}$: Blue, eCO${}_{2}$: Red). Subfigures (**A**–**F**): [model][data][prediction type]; training model: Final model fit on training data; full model: Final model fit on full data; fixed year model: See Table 6; Data: Training, test, full (test + training); with/no GE: With or without group-level effects.

**Figure A8:**
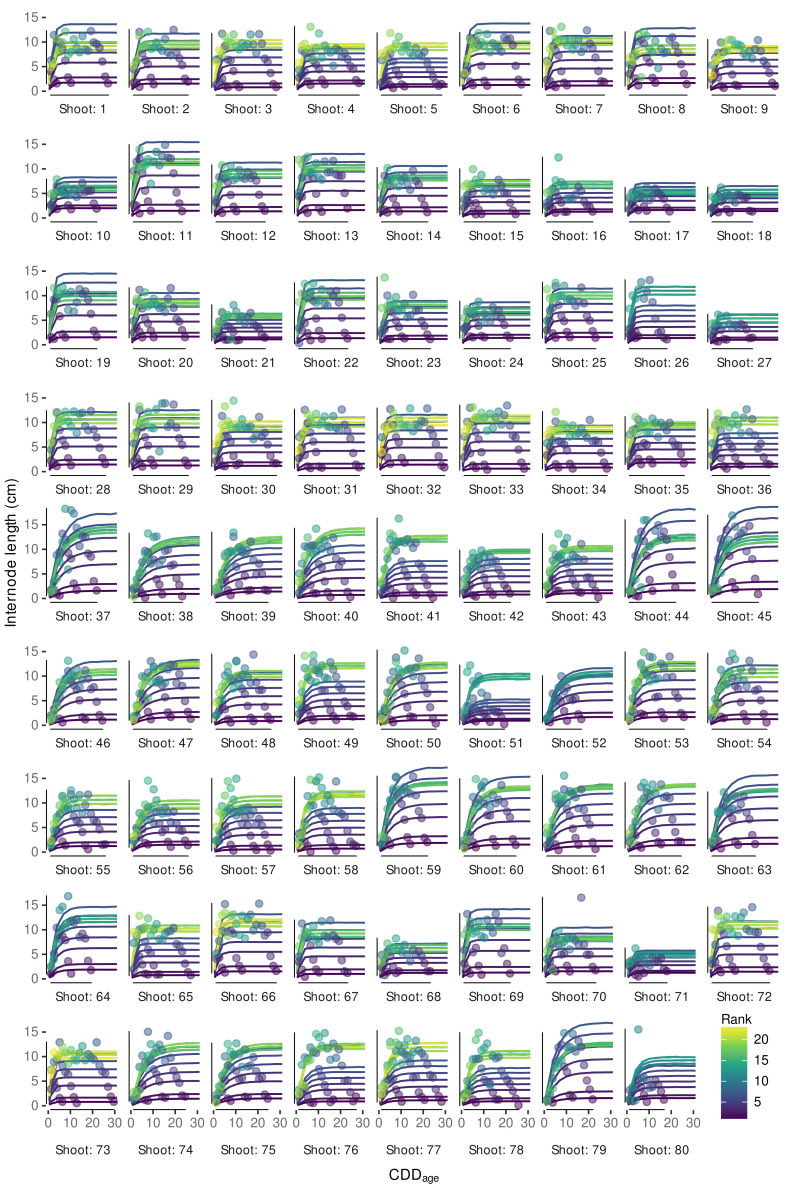
Model predictions (—) of internode length per shoot and rank using the final model with the full data set and all shoot’s group-level information in the predictions (measurement data (∘)).

For a more detailed analysis on the predictive capabilities of the Bayesian models, we compared various model predictions with the corresponding measurement data-points, while considering the modeled group-level effects or not. Comparing the different predictions of the final model fitted with training data only (Figure 14A–C) the estimated RMSE only based on the average (point) predictions is generally better than the full Bayesian RMSE provided in Table 6 and more comparable to the frequentist RMSE (Table A3). Considering the group-level effects in the predictions on training data clearly improved the point-wise performance measures RMSE, MAE and bias (Figure 14A vs. Figure 14B). Especially internode lengths above approximately 10 cm were underrepresented in average predictions not using group-level information. Step-wise removing group-level effects revealed that this missing of longer internodes was linked to the group-level effects at the single shoot level (data not shown). This indicates a high variability between single shoots. How well this variability could be captured by the final model that was fitted on the full data set can be seen in Figure A8, where predictions of internode growth curves over ${\mathrm{CDD}}_{\mathrm{age}}$ for each of the 80 shoots and all its internodes (ranks) are compared to measurement data.

For example, the full data set included shoots with, in general, shorter internodes (e.g., shoots 10, 17 or 71) and shoots with slightly delayed growth (e.g., shoots 52 or 79) indicated by a less steep slope (Figure A8). However, as we allowed all parameters to vary between shoots by group-level effects modeling, we see how singular outliers (e.g., shoot 80) did not distort the single internode behavior, as the hierarchical model counteracts by shrinkage towards a global mean [130]. Thus, the model benefits from partitioning sources of variation and shrinkage leading to more accurate and hence robust estimates of parameters [130,131].

Test data predictions not considering group-level effects do not fall off compared to training data predictions when comparing point-wise performance measures (Figure 14B vs. Figure 14C). A similar pattern of point-predictions (averages) not covering the full range is present. For the model out-of-sample performance, test data cannot be predicted considering group-level effects, as, for example, no shoot of the test data set has been present in the training data, thus no group-level effect has been estimated for this shoot. Random sampling from group-level effects would not be appropriate here, as this would only apply to a comparison of populations of shoots rather than to this point-pair based performance. Incorporating the fixed year effect (Figure 14C vs. Figure 14D) did not improve model predictive performance, here regarding test data, as already indicated by the LOOIC comparison in Section 3.4.1.

In the next step we use samples from the group-level effects to incorporate between-shoot variability back into a simulation model (see Section 2.10.3 and Section 3.4.3) that will allow capturing this natural variability in a stochastic FSP model. However, at this stage we only deciphered how average predictions and predictions with group-level effects affect model performance measures especially on the respective training data (i.e., training data set or full data set).

#### 3.4.3. Variability in Internode Length Simulations

**Figure 15:**
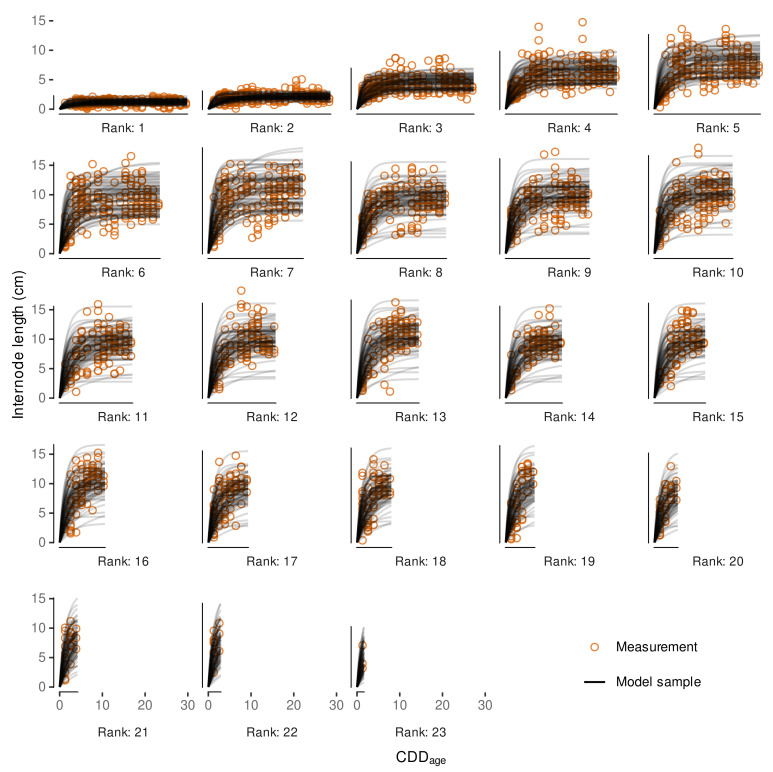
Model simulation of 80 different shoots and their internode length development by rank using 80 samples from the posterior distribution covering the time frame of the observations of approximately CDD = 30 (cumulative development days). The *x*-axis describes the age of each internode in CDD (${\mathrm{CDD}}_{\mathrm{age}}$).

**Figure A9:**
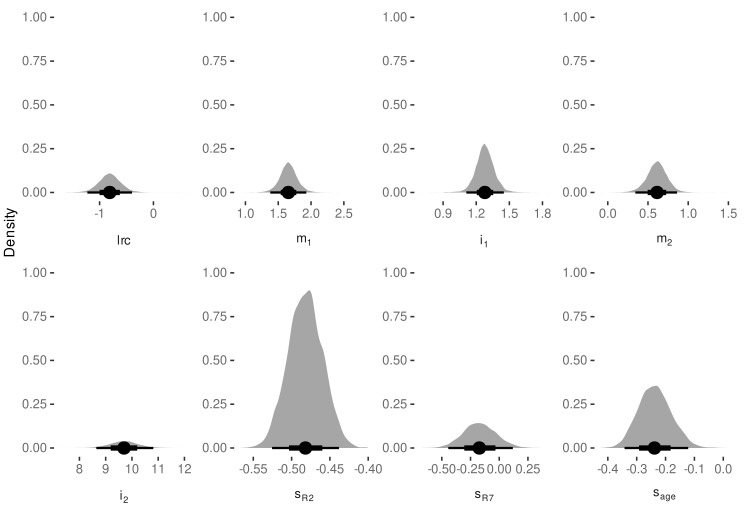
Posterior distributions of the main model coefficients for the final internode length model fitted on the full data set.

**Figure A10:**
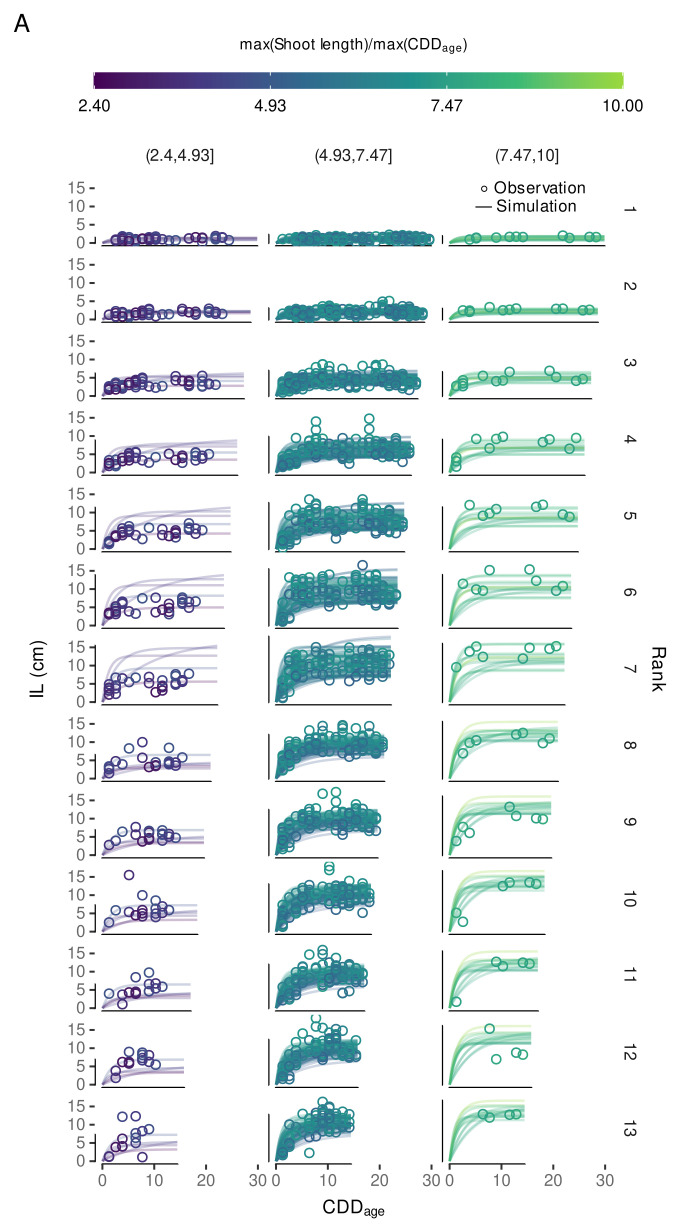

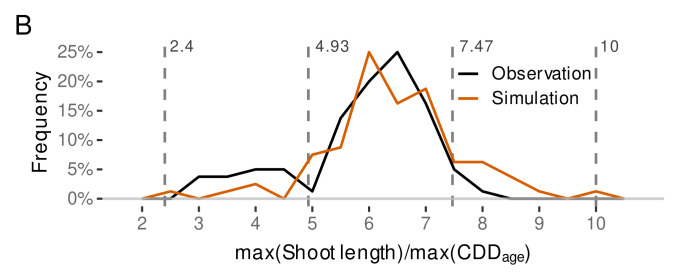
(**A**) Model simulation of 80 different shoots and their internode length development by ranks (rank ≤13) using samples from the posterior distribution including group-level effects covering the time frame of the observations of approximately CDD = 30 (cumulative development days). The x-axis describes the age of the internode in CDD (${\mathrm{CDD}}_{\mathrm{age}}$). Columns devise the relative max. shoot the length ($\max\left(\mathrm{shoot}\;\mathrm{length}\right)/\max\left({\mathrm{CDD}}_{\mathrm{age}}\right)$) into three different classes. (**B**) Frequency of relative shoot the length in the observation and the simulation data set.

Finally, we want to focus on how these modeling results could be transferred into a FSP model. Therefore, we rely on the final model fitted to the full data set to carry as much information as possible. We have shown that predictions with group-level effects perform best on the data used for model fitting (Figure 14E vs. Figure 14F) as they can capture the observation that individual shoots can be very different from each other. Thus, especially in a Bayesian analysis including the full data set into the final model, not only increases robustness in average parameter estimates, at the same time it enriches the posterior samples at group-level that can be used to represent natural variability. Now, each sampled parameter set (lrc, $m_{1}$, $i_{1}$, $m_{2}$, $i_{2}$, $s_{R2}$, $s_{R7}$) from the joint posterior distribution could be used to model the growth of an average shoot, more precisely, the growth of its internodes. To enrich these robust estimates of plausible average shoots the information captured in the group-level effect estimates can be considered. So we can reinsert natural variability by sampling from the corresponding posterior estimates for the group-level effects and adding them to the fixed effects (cf. Figure A9). Similar to basic predictions from frequentist models residual errors will not be transferred into simulations, but, as already discussed in the introduction, in a Bayesian analysis we are not limited to a single estimate for each parameter. As the age correction term was only relevant for corrections related to measurement uncertainties, it is removed from the simulation model by fixing it to $s_{\mathrm{age}}=0$. In this way, we simulated 80 different shoots by sampling parameter sets from the 12,000 stored posteriors samples of the model output. When comparing the overall fit of these predictions we can see a good agreement with the magnitude and, more importantly, also with the variability of the measurement data (Figure 15). At this stage we no longer compare point-wise predictions, where such incorporation of randomness would be disturbing; here, we target simulating a plausible population of shoot internode developments that could be used in a stochastic FSP model. In contrast to the the average predictions without considering the group-level effects (Figure 14F), these simulations do also cover internodes with length well above 10 cm. Thus, the Bayesian model results provide a vast enrichment in model output variability compared to only using the single point estimate parameter set that we would have ended up with when applying a frequentist approach, only. For example, we were able to successfully simulate different shoot length classes with similar frequency as in the measured data (Figure A10B). Classes were derived by calculating a normalized shoot length considering the total age of the shoot ($\max\left(\mathrm{shoot}\;\mathrm{length}\right)/\max\left({\mathrm{GDD}}_{\mathrm{age}}\right)$) that was split into three classes (see Figure A10A; data only shown for R≤13). In this single sample the results indicate a few more slightly longer shoots in the simulation than in the observation.

#### 3.4.4. External Validation Based on Shoot Length Ranges

**Figure 16:**
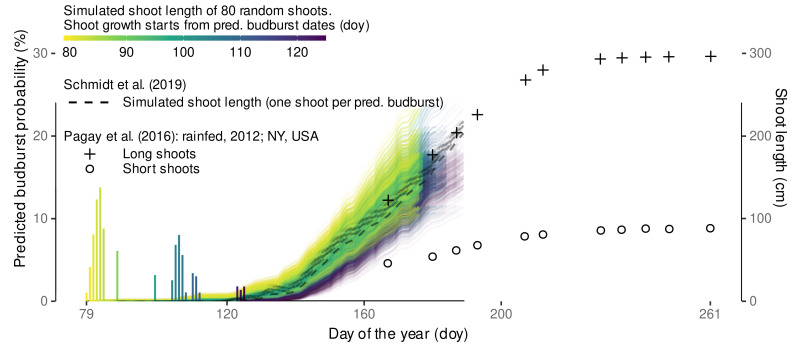
Shoot length model predictions from this study (80 random shoots per budburst data) and using the model from Schmidt et al. [12] (one shoot per predicted budburst date) compared to average measurements of short and long (rainfed) shoots from Pagay et al. (Figure 2a [115]) at Geneva, NY, USA. The predicted budburst probability for this studies budburst model is indicated and transported in the transparency of the line plots. Measurement data was extracted from the original figure using Webplotdigitizer [114].

To validate whether our model captures variability in shoot length observed in other Riesling vineyards, simulation of shoot length, i.e., the sum of internode lengths at a given time, were compared to experimental data on the development of short and long shoots selected from a drought stress experiment [115]. Hence, instead of quantifying errors of point estimates, we would rather point to the power of the Bayesian calibration to map natural variability in predictions. The simulation relies on all developed models from this study; budburst, appearance rate and internode length. Comparing first only the model predictions of this study to measurement data (Figure 16), the predictions are more close to the long shoot observations, but also include shoots tending towards the short shoot observations. We also added simulations with the respective model components from Schmidt et al. [12] (see Section 2.10.3) to this comparison. The simulations with this model are also closer to the longer shoot measurement with less variability, hence not coming that close to the short shoots, as the variability in this model is limited to budburst date variation. Thus, this model comparison suggests that both model predictions could over-predict shoot length to a certain extent; however, considering the described differences in vineyard setup and planting material (see Section 2.10.3), this should not be overinterpreted. Both, the rather low-vigor rootstock as well as the higher shoot load in Pagay et al. [115] might explain a bias towards a somewhat lower shoot growth rate compared to the Geisenheim VineyardFACE experiment. In addition, weak shoots are preferentially removed in the course of shoot thinning, limiting the presence of short shoots in our data set. However, the novel model better mimics the natural diversity of short and long shoots.

#### 3.4.5. Local, Independent Validation Using Shoot Length Data from 2020 Season

**Figure 17:**
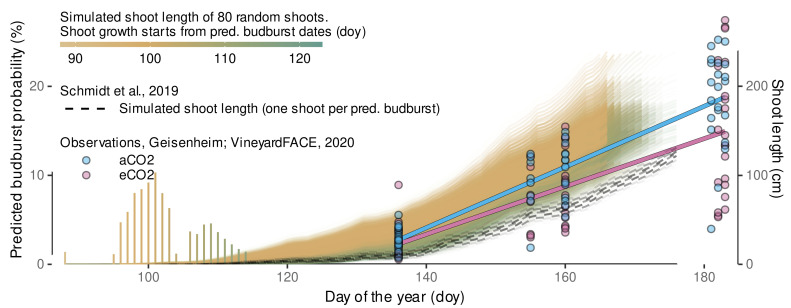
Shoot length model predictions for Geisenheim 2020 compared to measurements from the VineyardFACE facility in 2020. Solid, red/blue colored lines show simple linear regression results for the observations per treatment (aCO${}_{2}$, eCO${}_{2}$). The predicted budburst probability for this studies budburst model is indicated and transported in the transparency of the line plots.

**Figure A11:**
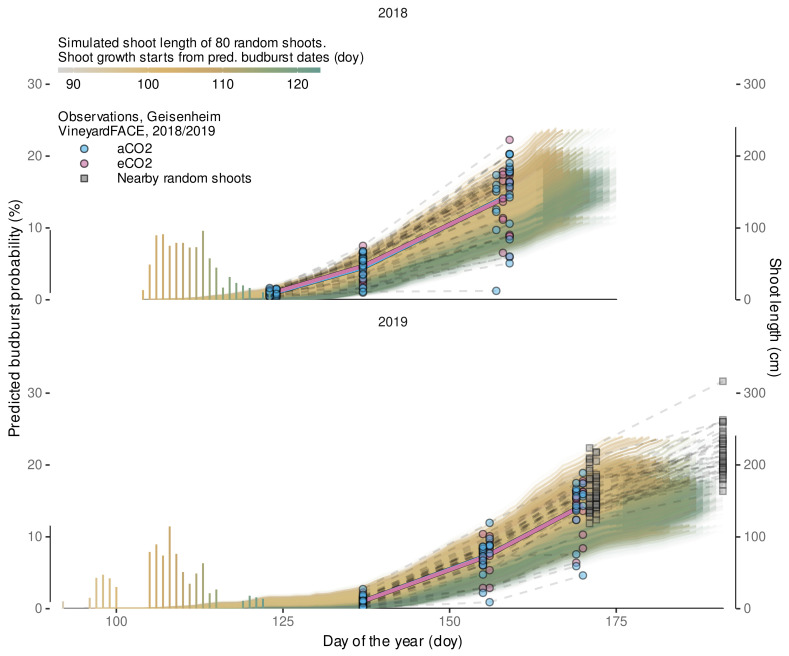
Shoot length model predictions for Geisenheim 2018 and 2019 compared to measurements from the VineyardFACE facility in the same years. Dashed lines connect data points from the same shoot. Solid, red/blue colored lines show simple linear regression results for the observations per treatment (aCO${}_{2}$, eCO${}_{2}$). The predicted budburst probability for this studies budburst model is indicated and transported in the transparency of the line plots. Additional observations include unpruned shoots (‘nearby random shoots’) from identical planting material grown outside of the FACE rings [116].

Shoot length data from VineyardFACE digitization measurements from a subsequent season (2020) were used for local validation. Comparing the model simulations of this study to the simulations using the model from Schmidt et al. [12], we found that the latter model seems to constantly under-predict shoot length, while the novel model is in line with the data at early stages, but over-predicts average shoot length later on (Figure 17). For quantitative comparison we calculated the weighted mean shoot length, with weighting by budburst frequency of that day (Table 7). As the regression lines for aCO${}_{2}$ and eCO${}_{2}$ drift apart, we split the comparison by treatment. Results show that the over-prediction of the new model is in the range of 2.6 cm to 14.4 cm for aCO${}_{2}$-shoots and between 6.9 cm and 34.3 cm for eCO${}_{2}$-shoots, while using the model from Schmidt et al. [12] under-predictions are in the range of 11.7 cm to 41.4 cm, with better performance for eCO${}_{2}$ shoots. Here, too, the measurement data shows a high natural variability that could satisfactorily be recovered by the novel model. The variability in the predictions using the Schmidt et al. [12]-model, where variability is based on budburst variability alone is too low. The trend towards longer shoots in the predictions using the here developed model, especially considering the eCO${}_{2}$ data, suggests that updating the model with additional data might be beneficial to increase future model performance and possibly differentiate between ambient and elevated vines. However, similar representation of the calibration years 2018 and 2019 (Figure A11) do support the model selection process, where no treatment effect could be estimated, as the simple regression lines for both treatments overlap in both years. Predictions of additional observations in 2019 from nearby random shoots of identical planting material that grew outside of the FACE rings [116] support the validity of the model calibration within that year (Figure A11). Inter-annual variability of grapevine vegetative growth is often caused by factors such as stress or source/sink ratio [132,133]. As these factors have not yet been integrated into the model, more data need to be integrated to capture these effects.

### 3.5. Future Work and Perspective Use Cases

In this case study, we extended model components of the functional-structural plant model Virtual Riesling aiming for realistic architectural representation of the grapevine variety Riesling grown in a FACE facility. The perspective goal is to use the advanced model in studies benefiting from a high level of architectural detail especially including intra-plant variability. Towards a full stochastical model further model components, e.g., for leaves, lateral shoots and related angles [12], need to be subjected to a similar Bayesian calibration as presented here. Moreover, an inclusion of further management practices, besides shoot positioning [12] and leaf removal [46], and their effects on growth are of interest to extend the applicability of such a virtual plant model. Bahr et al. [125] laid out a concept on necessary extension of grapevine functional-structural plant models to study grapevine berry sunburn, a recurring problem for viticulturists linked to climate change [134]. We expect the inclusion of variability in architectural components to provide a more concise picture of the microclimatic conditions within grapevine canopies [47,49], which would enable precise forecasts of sunburn occurrence, but also of other health and quality aspects related to microclimatic conditions. For studies aimed at simulations of future climatic conditions, the effects of elevated CO${}_{2}$ on grapevine architecture would also need to be further investigated [88].

## 4. Conclusions

The primary goal of this work was to advance the development of a fully stochastic virtual plant model for Riesling grapevine. In the course, we introduce the concepts and benefits of Bayesian calibration to a broad audience, using a case study on the effects of CO${}_{2}$ enrichment on grapevine (*Vitis vinifera* L. cv. Riesling) shoot development, starting from phenological data and digitized shoots with an empirical modeling concept based on temperature dependencies. Validation results have shown that model outputs were in good agreement with published or public data sets for budburst dates, as well as internode appearance and shoot growth rates. However, model reduction steps using information criteria revealed that we could not distinguish between elevated and atmospheric CO${}_{2}$ conditions in either phenology or primary shoot growth, with the considered enrichment corresponding to the CO${}_{2}$ level expected in 2050. This study further demonstrates that Bayesian calibration in combination with mixed models can realistically recover natural shoot growth variability in predictions. When implemented into a functional-structural plant model, we expect improvements for simulations where variability in microclimatic conditions is of relevance, e.g., in studies to predict berry sunburn occurrence, or other grape health and quality attributes in a virtual vineyard.

## Figures and Tables

**Table 1:** Linearization of phenological stages of grapevine between E-L stage 1 and stage 18 (ELSt), fixing stage 4 (budburst) and linearization of stages (ELSt${}_{\mathrm{linear}}$) depending on number of seperated leaves ($n_{\mathrm{leaves}}$).

$n_{\mathrm{leaves}}$						1	(2-)3	4	5	6	7	8	10	12	14
ELSt	1	2	3	**4**	5	7	9	11	12	13	14	15	16	17	18
ELSt${}_{\mathrm{linear}}$	2.00	3.75	3.875	**4.00**	5.00	6.50	8.50	9.50	10.50	11.50	12.50	13.50	15.50	17.50	19.50

**Table 2:** Public data sources used in the validation of the phenology model.

Location	Season	Stage	Source
Neustadt an der Weinstraße, Germany	2003–2021	’Austrieb’ (budburst), ‘Blühbeginn’ (beginning of flowering)	https://www.dlr-rheinpfalz.rlp.de/Internet/global/themen.nsf/2eca2af4a2290c7fc1256e8b005161c9/8096dedb652c43cbc12571b00048fe49?OpenDocument (accessed on 27 August 2021)
Zeltingen-Rachtig, Germany	2013–2016	budburst (BBCH 09 [110]), beginning of flowering	Molitor et al. [111], Suppl. Mat: https://ojs.openagrar.de/index.php/VITIS/article/view/8462/8625 (accessed on 7 February 2022)
Remich, Luxembourg	1993–2015	budburst (BBCH 09 [110])	Molitor and Keller (Table 2 [112])
Geisenheim, Germany	1990–2009	budburst	Stoll et al. (Figure 1 [14])

**Table 3:** Results of Pareto optimization following the first and second grid search iteration on how often a cardinal temperature triplet was associated with a Pareto optimal solution ($n_{\mathrm{opt}}$) from a total of 219 Pareto optimal solutions. Data on iteration 1 includes all 219 optima, while iteration 2 is limited to solutions with $n_{opt}\geq 4$.

Iteration 1				Iteration 2			
$T_{\mathrm{base}}$	$T_{\mathrm{opt}}$	$T_{\mathrm{upper}}$	$n_{\mathrm{opt}}$	$T_{\mathrm{base}}$	$T_{\mathrm{opt}}$	$T_{\mathrm{upper}}$	$n_{\mathrm{opt}}$
11	19	25	67	10.8	19.0	24.7	49
9	19	21	37	11.8	18.7	24.1	30
12	16	35	28	10.9	19.1	25.3	19
15	17	22	24	11.8	18.8	24.1	15
11	19	24	23	10.6	19.3	25.3	14
−8	21	24	15	11.5	18.7	24.2	13
−38	22	25	9	10.6	19.8	25.4	8
12	15	50	4	11.3	19.1	24.4	6
−11	20	22	4	10.5	19.5	25.0	5
15	17	21	2	11.9	18.7	24.3	5
8	20	24	2	11.9	18.7	24.2	5
11	19	23	2	11.6	18.9	24.2	4
9	19	22	1				
4	21	25	1				

**Table 4:** Model result from step-wise model reduction: Starting from a full model (including year effect and interaction between treatment (trt) and linearized E-L stage (ELSt_linear_; Equation (5))) to a reduced, final model only considering the stage, plus the downstream final exGaussian model. Performance measures (leave-one-out cross-validation information criterion and standard error (LOOIC (SE)), Bayesian RMSE and R2) and parameter estimates and their probability of direction (pd) are provide for each step.

Model	LOOIC [SE]	RMSE [95% HDI]	RMSE (test) [95% HDI]	R2 [95% HDI]	Parameter	Estimate [Q2.5, Q97.5]	pd (%)
full	9234.46 [95.41]	3.188 [3.0925, 3.2912]	3.0593 [2.8977, 3.2232]	0.8893 [0.8856, 0.8929]	ELSt_linear_	1.2892 [1.259, 1.3203]	100.00
full					Intercept	2.5436 [1.9893, 3.082]	100.00
full					trt_eCO2_	−0.0481 [−0.708, 0.6182]	56.43
full					trt_eCO2_:ELSt_linear_	−4 $\times 10^{-4}$ [−0.0402, 0.0406]	50.50
full					year_2019_	−0.0237 [−0.4223, 0.3781]	54.86
no interaction	9233.05 [95.37]	3.1866 [3.0892, 3.2842]	3.0593 [2.9053, 3.2345]	0.8893 [0.8857, 0.8931]	ELSt_linear_	1.289 [1.2682, 1.3099]	100.00
no interaction					Intercept	2.5352 [2.0315, 3.0276]	100.00
no interaction					trt_eCO2_	−0.0479 [−0.6024, 0.5175]	58.26
no interaction					year_2019_	−0.0178 [−0.4036, 0.3833]	54.05
no year	9232.04 [95.39]	3.1856 [3.0897, 3.2842]	3.0576 [2.8999, 3.2182]	0.8893 [0.886, 0.8933]	ELSt_linear_	1.2891 [1.2685, 1.3101]	100.00
no year					Intercept	2.5262 [2.0715, 2.983]	100.00
no year					trt_eCO2_	−0.0518 [−0.5796, 0.4729]	59.39
final	9232.64 [95.39]	3.1817 [3.0891, 3.2836]	3.0553 [2.8938, 3.2123]	0.8893 [0.8857, 0.8928]	ELSt_linear_	1.2893 [1.269, 1.3092]	100.00
final					Intercept	2.5006 [2.1734, 2.8311]	100.00
final (exGaussian)	8775.62 [79.81]	3.2624 [3.1183, 3.3996]	3.1466 [2.9118, 3.3836]	0.8789 [0.875, 0.8829]	ELSt_linear_	1.2539 [1.2345, 1.2736]	100.00
final (exGaussian)					Intercept	2.8502 [2.5594, 3.1455]	100.00
final (exG., full data)	11,288.56 [90.97]	3.1998 [3.0805, 3.3182]	—	0.8887 [0.8853, 0.8919]	ELSt_linear_	1.2577 [1.241, 1.2741]	100.00
final (exG., full data)					Intercept	2.7789 [2.4794, 3.0868]	100.00

**Table 5:** Model results of internode appearance rate estimation: Estimated fixed effects comparing the full model (including year and treatment effect; Table 5) and the reduced, final model with Gaussian likelihood only considering CDD (leave-one-out cross-validation information criterion and standard error (LOOIC (SE)). (rm: Remove; GE: Group-level effect.)

Model	LOOIC [SE]	RMSE [95% HDI]	RMSE (Test) [95% HDI]	R2 [95% HDI]	Parameter	Estimate [Q2.5, Q97.5]	pd (%)
full	642.61 [17.97]	2.3803 [2.1842, 2.5924]	2.5867 [2.279, 2.8805]	0.9751 [0.9707, 0.9791]	Intercept	−6.7827 [−7.8887, −5.7296]	100.00
full					CDD	0.7747 [0.7372, 0.8118]	100.00
full					CDD:trt${}_{eCO2}$	0.0087 [−0.0418, 0.0593]	63.65
full					trt${}_{eCO2}$	−0.0139 [−1.1569, 1.1765]	51.11
full					year${}_{2019}$	−0.268 [−1.2436, 0.7435]	71.44
rm trt interaction	639.82 [17.89]	2.3721 [2.178, 2.567]	2.574 [2.2815, 2.873]	0.9752 [0.9708, 0.9792]	Intercept	−6.8255 [−7.8867, −5.7655]	100.00
rm trt interaction					CDD	0.7792 [0.7533, 0.8041]	100.00
rm trt interaction					trt${}_{eCO2}$	0.0582 [−1.0514, 1.1574]	54.84
rm trt interaction					year${}_{2019}$	−0.2694 [−1.2459, 0.7285]	71.25
rm year	638.34 [17.53]	2.3748 [2.1999, 2.5562]	2.5915 [2.3077, 2.8755]	0.9753 [0.9711, 0.9793]	Intercept	−6.9295 [−7.8836, −6.023]	100.00
rm year					CDD	0.7794 [0.7537, 0.8054]	100.00
rm year					trt${}_{eCO2}$	0.0427 [−1.0245, 1.124]	53.00
rm trt	638.99 [17.8]	2.3521 [2.1713, 2.5334]	2.5572 [2.2669, 2.8495]	0.9752 [0.9709, 0.9792]	Intercept	−6.7851 [−7.6507, −5.9236]	100.00
rm trt					CDD	0.7797 [0.7541, 0.8059]	100.00
rm trt					year${}_{2019}$	−0.2686 [−1.2425, 0.7031]	71.39
add year GE	640.27 [17.92]	2.4214 [2.1622, 2.7362]	2.6286 [2.2688, 3.0283]	0.9752 [0.9712, 0.9793]	Intercept	−6.9483 [−8.3861, −5.6148]	100.00
add year GE					CDD	0.7798 [0.7539, 0.8051]	100.00
final	640.12 [17.99]	2.3589 [2.2037, 2.5365]	2.5789 [2.3162, 2.8699]	0.9753 [0.9712, 0.9793]	Intercept	−6.9059 [−7.6626, −6.1822]	100.00
final					CDD	0.7789 [0.7533, 0.8051]	100.00
final (full data)	832.6 [20.89]	2.4104 [2.2682, 2.5492]	−−−	0.9745 [0.9707, 0.978]	Intercept	−6.921 [−7.5665, −6.2985]	100.00
final (full data)					CDD	0.7784 [0.7547, 0.8018]	100.00

**Table 6:** Model performance of Bayesian generalized non-linear mixed models for internode length depending on CDD${}_{\mathrm{age}}$.

Model	LOOIC [SE]	RMSE [95% HDI]	RMSE (Test) [95% HDI]	R2 [95% HDI]	Parameter	Estimate [Q2.5, Q97.5]	pd (%)
fixed trt	6731.24 [101.24]	3.8366 [2.1598, 7.2981]	3.9045 [2.2945, 7.3602]	0.8466 [0.8396, 0.8536]	$s_{\mathrm{age}}$	−0.2172 [−0.3351, −0.0854]	99.92
fixed trt					$s_{R2}$	−0.48 [−0.5296, −0.4313]	100.00
fixed trt					$s_{R7}$	−0.3748 [−0.6671, −0.0708]	99.11
fixed trt					$i_{1,\mathrm{aCO}2}$	1.2515 [0.537, 1.8146]	100.00
fixed trt					$i_{1,\mathrm{eCO}2}$	1.2428 [0.5443, 1.8105]	100.00
fixed trt					$i_{2,\mathrm{aCO}2}$	10.0877 [8.2055, 11.9674]	100.00
fixed trt					$i_{2,\mathrm{eCO}2}$	9.6182 [7.7617, 11.5094]	100.00
fixed trt					lrc${}_{\mathrm{aCO}2}$	−0.797 [−1.6328, −0.0273]	97.77
fixed trt					lrc${}_{\mathrm{eCO}2}$	−0.9274 [−1.7598, −0.1525]	99.00
fixed trt					$m_{1,\mathrm{aCO}2}$	1.6845 [1.0109, 2.3235]	100.00
fixed trt					$m_{1,\mathrm{eCO}2}$	1.647 [0.9916, 2.2534]	100.00
fixed trt					$m_{2,\mathrm{aCO}2}$	0.6347 [0.1027, 1.1799]	98.89
fixed trt					$m_{2,\mathrm{eCO}2}$	0.5439 [0.0166, 1.0948]	97.78
fixed year	6731.92 [101.6]	3.5725 [2.0063, 6.9003]	3.6335 [2.1216, 6.9794]	0.8467 [0.8395, 0.8536]	$s_{\mathrm{age}}$	−0.2178 [−0.3378, −0.0841]	99.92
fixed year					$s_{R2}$	−0.4795 [−0.529, −0.4287]	100.00
fixed year					$s_{R7}$	−0.3763 [−0.6666, −0.0714]	99.15
fixed year					$i_{1,\mathrm{year}2018}$	1.3029 [1.0787, 1.5259]	100.00
fixed year					$i_{1,\mathrm{year}2019}$	1.2628 [1.0168, 1.5155]	100.00
fixed year					$i_{2,\mathrm{year}2018}$	8.1287 [7.3206, 8.9449]	100.00
fixed year					$i_{2,\mathrm{year}2019}$	11.4742 [10.5318, 12.4614]	100.00
fixed year					lrc${}_{\mathrm{year}2018}$	−0.283 [−0.5315, −0.0391]	98.65
fixed year					lrc${}_{\mathrm{year}2019}$	−1.3557 [−1.5916, −1.1194]	100.00
fixed year					$m_{1,\mathrm{year}2018}$	1.5069 [1.1818, 1.8159]	100.00
fixed year					$m_{1,\mathrm{year}2019}$	1.8811 [1.5457, 2.2236]	100.00
fixed year					$m_{2,\mathrm{year}2018}$	0.7489 [0.4476, 1.062]	100.00
fixed year					$m_{2,\mathrm{year}2019}$	0.2729 [−0.1296, 0.6765]	91.47
no fixed	6737.69 [101.75]	3.8481 [2.1888, 7.2349]	3.8924 [2.2786, 7.2188]	0.8465 [0.8395, 0.8538]	$s_{\mathrm{age}}$	−0.2179 [−0.3402, −0.086]	99.88
no fixed					$s_{R2}$	−0.4796 [−0.5293, −0.4297]	100.00
no fixed					$s_{R7}$	−0.3766 [−0.6703, −0.0737]	99.26
no fixed					$i_{1}$	1.2663 [0.5514, 1.8706]	100.00
no fixed					$i_{2}$	9.8205 [7.844, 11.8148]	100.00
no fixed					lrc	−0.8415 [−1.7012, −4e−04]	97.50
no fixed					$m_{1}$	1.6856 [1.1053, 2.2669]	100.00
no fixed					$m_{2}$	0.5593 [0.0307, 1.0996]	97.91
no year = final	6736.88 [101.64]	2.7603 [2.4947, 3.0985]	2.8545 [2.5506, 3.1883]	0.8462 [0.8389, 0.8533]	$s_{\mathrm{age}}$	−0.2185 [−0.3383, −0.0863]	99.85
no year = final					$s_{R2}$	−0.4779 [−0.528, −0.4284]	100.00
no year = final					$s_{R7}$	−0.3627 [−0.6528, −0.0528]	98.81
no year = final					$i_{1}$	1.2905 [1.1, 1.4792]	100.00
no year = final					$i_{2}$	9.7018 [8.5788, 10.8534]	100.00
no year = final					lrc	−0.8119 [−1.2259, −0.3988]	99.96
no year = final					$m_{1}$	1.6843 [1.4069, 1.9595]	100.00
no year = final					$m_{2}$	0.5879 [0.2818, 0.8683]	99.89
final (full data)	8436.07 [112.44]	2.7954 [2.5169, 3.1104]	2.7953 [2.5242, 3.1178]	0.8473 [0.8412, 0.8535]	$s_{\mathrm{age}}$	−0.2368 [−0.342, −0.1215]	99.98
final (full data)					$s_{R2}$	−0.482 [−0.5255, −0.4381]	100.00
final (full data)					$s_{R7}$	−0.1699 [−0.4437, 0.1185]	88.13
final (full data)					$i_{1}$	1.2795 [1.1104, 1.4503]	100.00
final (full data)					$i_{2}$	9.6989 [8.6432, 10.8139]	100.00
final (full data)					lrc	−0.812 [−1.227, −0.3979]	99.87
final (full data)					$m_{1}$	1.6552 [1.3787, 1.9278]	100.00
final (full data)					$m_{2}$	0.6104 [0.3429, 0.8614]	99.98

**Table 7:** Average shoot length from observation (Geisenheim, VineyardFACE 2020) and model simulations (Schmidt et al. [12]) and this study) for three dates and the two treatments (aCO${}_{2}$, eCO${}_{2}$).

		Observation	Schmidt et al., 2019		This Study	
Doy	Treatment	${\bar{\mathrm{SL}}}_{\mathrm{obs}}$ (cm)	$\bar{\mathrm{SL}}$ (cm)	$\Delta \bar{\mathrm{SL}}$ (cm)	$\bar{\mathrm{SL}}$ (cm)	$\Delta \bar{\mathrm{SL}}$ (cm)
136	eCO${}_{2}$	25.2	10.0	−15.1	32.1	6.9
136	aCO${}_{2}$	29.5	10.0	−19.5	32.1	2.6
155	eCO${}_{2}$	68.0	56.3	−11.7	101.1	33.1
155	aCO${}_{2}$	89.3	56.3	−33.0	101.1	11.8
160	eCO${}_{2}$	84.9	63.3	−21.6	119.2	34.3
160	aCO${}_{2}$	104.8	63.3	−41.4	119.2	14.4

**Figure A4:**
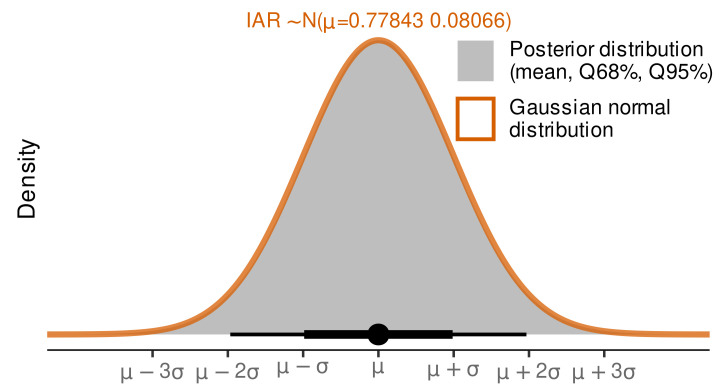
Density plus the mean and 68%/95% quantiles of the posterior distribution for the slope model coefficient (*n* = 8000 samples) compared to a normal distribution with same mean and standard deviation.

## Data Availability

The final models presented in this study are openly available in Schmidt, D., Kahlen, K., Bahr, C., and Friedel, M. (2022). Models from “Towards a stochastic model to simulate grapevine architecture: A case study on digitized Riesling vines considering effects of elevated CO_2_.”, osf.io/4cb8y at https://doi.org/10.17605/OSF.IO/4CB8Y.

## Abbreviations

The following abbreviations are used in this manuscript:

aCO${}_{2}$	ambient carbon dioxide
CO${}_{2}$	carbon dioxide
CDD	cumulative development days
doy	day of the year
eCO${}_{2}$	elevated carbon dioxide
ELPD	expected log predictive densit
FACE	free air carbon dioxide enrichment
FE	fixed effect
FSP model	functional-structural plant model
GE	group-level effect
HDI	highest density interval
LOOIC	leave-one-out cross-validation information criterion
MAE	mean absolute error
q	quantile
RMSE	root mean squared error

## Appendix A

### Appendix A.1. More on Phenology Modeling

Recently, Molitor et al. [24] developed a model called *UniPhen* covering the stages from bud swell to berry ripening that also tries to capture the effect of different grape cultivars grown under climate conditions from the Luxembourgish grapegrowing region. Its accuracy across all stages and cultivars allows predicting 82% of the observations within ±7 days. Their GDD accumulation of daily average air temperatures is regulated by three different threshold temperatures (lower, upper and heat threshold) and is calculated relative to the budburst date (BBCH 09) for Riesling cultivar. In a study of Leolini et al. [25] six phenological models were compared in their ability to predict budburst, especially comparing approaches that do or do not consider the endo-dormancy period, i.e., forcing or chilling/forcing approaches. They conclude that model accuracies are comparable for both approaches with root-mean-squared-errors (RMSE) of around 7–10 days, but models including the endo-dormancy period were less prone to the selected starting date for temperature accumulation. Calibration was based on data collected in different European regions and conducted separately for different cultivars [25]. Similar predictive accuracies were achieved in a recent study limited to north-west Spain where approx. 65–100% of observations fell within a range of ±6 days from the predicted dates of budburst and flowering using one model calibration across four cultivars [26]. Another aspect considered in phenology modeling is whether different threshold temperatures are necessary to predict different phenological stages. A study on phenology modeling of Chardonnay subjected to forced regrowth after heavy pruning and defoliation conduced by Prats-Llinàs et al. [27] found best accuracy when using different upper threshold temperatures for each considered phenological stage, while assuming a constant base temperature of 5 ${}^{\circ }C$ for all stages. Predicting berry maturity seemed to be less temperature dependent and hence the least accurate (RMSE of 8–9 days); thus, it is suggested to improve the model for berry maturity by including other factors such as crop load or water availability [27]. Their validation data included a control from natural growing conditions (no forced regrowth), implicitly suggesting similar threshold temperatures are applicable for both variants in early growing stages. As already partly addressed above, one of the aspects open for optimization is the estimation procedure of the GDD summation. For instance, the *UniPhen* [24] already includes a heat threshold temperature of 30 ${}^{\circ }C$ to implement a decelerating effect of above-optimum temperatures; however, note that concerning the use of average daily temperatures, this threshold is not too influential under current Central European climate conditions. While average daily temperatures of above 30 ${}^{\circ }C$ are not to be expected any time soon in Central Europe, hourly temperature averages of this magnitude are not uncommon during the summer growing season.

Where most GDD models for predicting phenological stages rely on one or a few summary statistics of the daily temperature course, i.e., the average, the minimum and the maximum temperature, de Cortázar-Atauri et al. [32] compared two models, where the daily sum is the sum of hourly contributions, but the temperature course of a day itself is a linear or sinusoidal model calculated from daily minimum and maximum temperatures. Improving growing degree day models is also an aspect in more general crop simulation research. For example, to consider a sub-daily non-linear behavior in the summation of GDD, Zhou and Wang [31] introduced a new method, where the daily temperature contribution is the result of an hourly thermal time estimation, with a non-linear response modeled by a beta-distribution function. This method proved to be superior to classical methods based on daily summary temperature measures in estimating various developmental stages of crops (corn and wheat), as well as to a model also considering hourly temperatures, but with linear response to deviations from the optimal temperature.

However, a similar concept had already been introduced by Wang and Engel [29] in form of a more general temperature response function that is scaled to values between 0 and 1. This approach was applied by de Cortázar-Atauri et al. [33] in grapevine phenological modeling, but using fixed lower and upper temperature limits of 0 ${}^{\circ }C$ and 40 ${}^{\circ }C$, respectively. Most recently, Zhu et al. [28] adapted this concept to calculate necessary development days, i.e., the number of days (24 h) at optimal temperatures, for specific grapevine phenological phases after budburst. Calibration focused on Sauvignon Blanc grapevines, but also included Chardonnay, Merlot, Pinot Noir and Pinot Gris. Estimated cardinal temperatures were optimized based on best prediction for flowering and véraison simultaneously. They rely on sinusoidal interpolations of hourly temperatures based on daily minimum and maximum temperatures [28,30]. For budburst a chilling-forcing approach was used, where the heat unit accumulation follows a simple linear growing degree days approach only depending on a base temperature. In their model calibration the base temperature was estimated to 0.008 ${}^{\circ }C$ for all varieties and differed from the estimated minimum temperature for the development after budburst, which was in the range of 0.2–3.9 ${}^{\circ }C$.

### Appendix A.2. More on Bayesian Model Calibration

Bayesian calibration approaches have been successfully applied in various fields. One of the first use-cases was the introduction of Bayesian model calibration in process-based forest models by Oijen et al. [2]. Integrating remote sensing data into Bayesian model calibration to simulate forest production was the aim of the study of Patenaude et al. [135]. They justify their choice of a Bayesian calibration over standard goodness-of-fit approaches by the enriched information on parameter and model output uncertainties. van Oijen et al. [136] applied Bayesian calibration for modeling growth of scots pine stands highlighting the effect of reduced model predictive uncertainties. Recently, Ovalle-Rivera et al. [137] was able to improve model accuracy by making use of a hierarchical Bayesian model calibration for a parameter-rich dynamic model for coffee agroforestry. Other applications Bayesian calibration, for example, go from maize yield predictions from empirical non-liner growth response functions [9], over accounting for temporal variability in growth models in the field of aquatic sciences [7] to considering uncertainties related even in astronomical estimations [138]. Moreover, improvements in modeling phenological development of maize were achieved with Bayesian calibration [79]. For simple models Ceglar et al. [79] found posterior distributions that included physiologically unrealistic values, suggesting that such model formulations might need to be revised. In a climate impact study of Ben Touhami and Bellocchi [139] it was found that the inclusion of prior information was beneficial for uncertainty estimates, even if these information came from different climate conditions. Blanc et al. [140] point out that a pitfall of Bayesian model calibration for functional-structural plant models is that with transferring uncertainties in model parameters repetitive simulation runs are necessary for robust estimates of output variables. To address this, they propose a surrogate modeling approach to substitute time consuming FSP model simulations with simpler models, such as Kriging models. It should be noted that repetitive simulations are not only necessary if Bayesian calibration is used, any randomness included in a model will require multiple runs [141].

**Table A1:** Number of phenology assessments per year, ring and day of the year.

		Day of the Year (Doy)								
Year	Ring	98	109	115	122	126	135	140	145	154
2018	aCO${}_{2}$[1]		60		60		48		60	
2018	aCO${}_{2}$[2]		40		60		44		60	
2018	aCO${}_{2}$[3]		51						52	
2018	eCO${}_{2}$[1]		60		56		38		60	
2018	eCO${}_{2}$[2]		49		60		51		60	
2018	eCO${}_{2}$[3]		60		46		37		60	
2019	aCO${}_{2}$[1]	60		60		60		29		60
2019	aCO${}_{2}$[2]	12		55		60		36		60
2019	aCO${}_{2}$[3]	24		49		60		39		45
2019	eCO${}_{2}$[1]	60		60		60		35		60
2019	eCO${}_{2}$[2]	60		53		60		36		60
2019	eCO${}_{2}$[3]	48		53		60		37		60

**Listing A1.** Codeblock of brms-formula for the final internode length model.

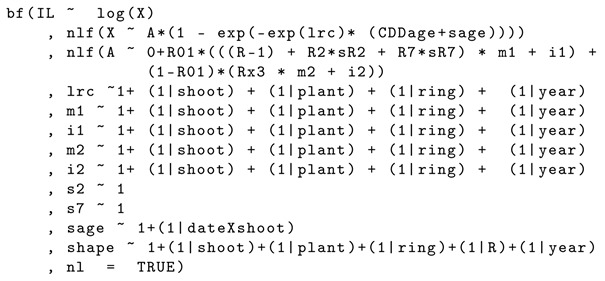

**Table A2:** Average internode length ($\bar{\mathrm{IL}}$) and number of observations per rank (*n*) and year for the training and test data set. Total number of observations (training/test): 2658 (2124/534) from 80 (64/16) different shoots.

	Year 2018				Year 2019			
	Training		Test		Training		Test	
Rank	n	$\bar{\mathrm{IL}}$	n	$\bar{\mathrm{IL}}$	n	$\bar{\mathrm{IL}}$	n	$\bar{\mathrm{IL}}$
1	108	1.25	27	1.21	84	1.06	21	1.12
2	108	2.03	27	1.92	83	2.01	21	1.73
3	107	3.96	27	3.83	82	4.35	20	3.84
4	97	5.34	24	5.32	81	5.71	19	5.11
5	82	6.60	20	7.06	77	6.43	19	5.72
6	72	8.53	19	8.32	66	8.39	18	6.92
7	69	8.86	17	10.10	59	9.70	15	8.89
8	64	6.92	15	8.05	56	9.49	14	8.83
9	54	7.46	14	7.92	53	9.17	13	9.90
10	41	8.44	12	8.63	52	9.16	13	9.79
11	37	7.61	9	8.42	49	8.66	12	8.02
12	36	8.67	9	8.54	46	9.04	12	8.34
13	36	9.18	9	9.65	41	8.88	10	8.91
14	36	8.23	9	8.28	34	8.31	7	10.66
15	35	9.18	8	9.32	28	8.98	7	9.49
16	32	10.13	8	9.77	25	7.84	7	8.69
17	32	8.62	8	9.20	24	6.67	6	5.98
18	27	9.63	7	8.03	19	5.59	5	6.02
19	20	10.18	4	12.78	17	3.87	5	4.42
20	18	7.75	4	10.59	10	3.35	3	4.41
21	14	7.20	4	10.17	2	1.25	2	3.18
22	9	6.70	2	10.20			1	2.45
23	2	3.59	1	7.08				

**Table A3:** Model performance of frequentist non-linear mixed models for internode length depending on CDD${}_{\mathrm{age}}$.

Model	AICc	RMSE	RMSE (Test)	Parameter	Estimate
trt	8125.19	2.19	2.34		
no trt	8115.96	2.19	2.30		
no trt; heteroscedasticity (fitted values)	7551.48	2.17	2.27		
no trt; heteroscedasticity (per rank)	7327.16	2.17	2.29		
trt; heteroscedasticity (fitted values and per rank)	7272.82	2.15	2.30		
no trt; heteroscedasticity (fitted values and per rank)	7263.05	2.16	2.28	$m_{1}$	1.55
				$i_{1}$	1.29
				$m_{2}$	0.45
				$i_{2}$	9.56
				lrc	−0.56
				$s_{\mathrm{age}}$	−0.56
				$s_{R2}$	−0.43
				$s_{R7}$	−0.49

**Table A4:** Prior input for Bayesian phenology models (FE = fixed effect, GE = group-level effect). No differentiation in priors within FE and GE, respectively.

Prior	Class
student_t(10, 0, 1)	FE
student_t(3, 12.6, 8.4)	Intercept
normal(0, 1)	GE
normal(0, 1)	GE
student_t(3, 0, 8.4)	sigma

**Table A5:** Prior input for Bayesian internode appearance rate models (FE = fixed effect, GE = group-level effect). No differentiation in priors within FE and GE, respectively.

Prior	Class
student_t(10, 0, 1)	FE
student_t(3, 9.5, 6.7)	Intercept
student_t(3, 0, 1)	GE
student_t(3, 0, 6.7)	sigma

**Table A6:** Prior input for Bayesian internode length models (FE = fixed effect, GE = group-level effect, dpar = distributional parameter, nlpar = non-linear parameter).

Prior	Class	Dpar	Nlpar	Bound
normal(0.6, 0.3)	FE		sage	
normal(1, 1)	FE		i1	<lower = 0>
normal(10, 2)	FE		i2	<lower = 0>
normal(−1, 1)	FE		lrc	
normal(1.5, 1)	FE		m1	<lower = 0>
normal(0.7, 0.5)	FE		m2	
normal(0, 0.5)	FE		sR2	
normal(0, 0.5)	FE		sR7	
student_t(3, 0, 2.5)	GE	shape		
student_t(3, 0, 2.5)	GE		sage	
normal(0, 1)	GE		i1	
normal(0, 1)	GE		i2	
normal(0, 0.5)	GE		lrc	
normal(0, 0.5)	GE		m1	
normal(0, 0.5)	GE		m2	
student_t(3, 0, 2.5)	GE	shape		
